# An Atlas of Altered Expression of Deubiquitinating Enzymes in Human Cancer

**DOI:** 10.1371/journal.pone.0015891

**Published:** 2011-01-25

**Authors:** Chiara Luise, Maria Capra, Maddalena Donzelli, Giovanni Mazzarol, Maria Giovanna Jodice, Paolo Nuciforo, Giuseppe Viale, Pier Paolo Di Fiore, Stefano Confalonieri

**Affiliations:** 1 IFOM, Fondazione Istituto FIRC di Oncologia Molecolare, Milan, Italy; 2 Istituto Europeo di Oncologia, Milan, Italy; 3 Dipartimento di Medicina, Chirurgia e Odontoiatria, Università degli Studi di Milano, Milan, Italy; Karolinska Institutet, Sweden

## Abstract

**Background:**

Deubiquitinating enzymes (DUBs) are proteases that process ubiquitin (Ub) or ubiquitin-like gene products, remodel polyubiquitin(-like) chains on target proteins, and counteract protein ubiquitination exerted by E3 ubiquitin-ligases. A wealth of studies has established the relevance of DUBs to the control of physiological processes whose subversion is known to cause cellular transformation, including cell cycle progression, DNA repair, endocytosis and signal transduction. Altered expression of DUBs might, therefore, subvert both the proteolytic and signaling functions of the Ub system.

**Methodology/Principal Findings:**

In this study, we report the first comprehensive screening of DUB dysregulation in human cancers by in situ hybridization on tissue microarrays (ISH-TMA). ISH-TMA has proven to be a reliable methodology to conduct this kind of study, particularly because it allows the precise identification of the cellular origin of the signals. Thus, signals associated with the tumor component can be distinguished from those associated with the tumor microenvironment. Specimens derived from various normal and malignant tumor tissues were analyzed, and the “normal” samples were derived, whenever possible, from the same patients from whom tumors were obtained. Of the ∼90 DUBs encoded by the human genome, 33 were found to be expressed in at least one of the analyzed tissues, of which 22 were altered in cancers. Selected DUBs were subjected to further validation, by analyzing their expression in large cohorts of tumor samples. This analysis unveiled significant correlations between DUB expression and relevant clinical and pathological parameters, which were in some cases indicative of aggressive disease.

**Conclusions/Significance:**

The results presented here demonstrate that DUB dysregulation is a frequent event in cancer, and have implications for therapeutic approaches based on DUB inhibition.

## Introduction

The post-translational modification of proteins by mono- or poly-ubiquitination is critical for the regulation of protein stability, activity and interactions. Through the modulation of these target protein properties, ubiquitination controls several cellular programs, including signal transduction, vesicular transport, transcription, apoptosis, chromatin remodeling, and DNA repair [1]–[7]. Similar to other covalent modifications, such as phosphorylation or methylation, ubiquitination is reversible. Approximately 100 deubiquitinating enzymes (DUBs) are encoded by the human genome, of which 90 appear to be expressed [8]. These enzymes cleave the isopeptide linkage between the protein substrate and the ubiquitin (Ub) residue, thereby terminating Ub-dependent signaling. DUBs belong to the superfamily of peptidases, specifically to the cysteine- and metallo-peptidase families. On the basis of their Ub-protease domain, the cysteine-peptidase DUBs may be further organized into four subclasses: Ub carboxyl-terminal hydrolases, families 1 (UCH) and 2 (USP) [9], ovarian tumor-like (OTU or OTUBIAN) proteases [10], [11], and the Machado-Joseph disease (MJD or MACHADO) proteases [12]. In addition, one class of DUB metallo-enzymes has been described: the JAB1/MPN/Mov34 (JAMM) family [13].

DUBs participate in the regulation of several biological functions. Some DUBs have been found in complex with the proteasome, where their function is required for protein degradation and Ub recycling [14], [15]. In other cases, DUBs are involved in remodeling the Ub content of target proteins, a mechanism referred to as Ub-editing. This process might be involved in the rescuing of erroneously ubiquitinated proteins from proteasomal degradation, or in the fine modulation of the amount and type of Ub chains linked to particular substrates [16]. Finally, and not surprisingly given the vast involvement of the Ub system in intracellular signaling, virtually every aspect of cell regulation is intersected by DUBs, including regulation of transcription, chromatin remodeling, intracellular vesicular trafficking, DNA repair, cell cycle progression, apoptosis, and signal transduction kinase cascades (for recent reviews see [17], [18]).

Subversion of DUBs might, therefore, alter both the proteolytic and signaling functions of the Ub system. This is predicted to affect cellular homeostasis and, in certain circumstances, to promote cellular transformation. Indeed, both oncogenic and tumor suppressor roles have been proposed for a number of DUBs [18], [19], leading to the concept that they might represent attractive targets for novel cancer therapies ([20], [21] and references therein). Thus, a better understanding of the functional roles of DUBs in cancer might have important consequences for cancer treatment, especially in light of recent advances in the development of DUB-specific small molecule inhibitors [22]. However, understanding the exact role of DUBs in “real” cancers is complicated by the fact that DUBs have multiple substrates. Thus, an atlas of DUB alterations in human cancer might provide an important tool to direct future pharmacological developments. At the genetic level, mutations or rearrangements/translocations of DUBs seem rare (with the important caveat that the issue has not been extensively investigated). Conversely, alterations in the levels of expression appear to be more frequent (for recent reviews of cancer-related DUBs, see [18], [19]). Here, we report the first comprehensive screening of alterations in DUB expression in nine human cancers. This study represents the first step towards the compilation of a systematic catalog of DUB dysregulation in cancer.

## Results

### Study design

**Figure 1:**
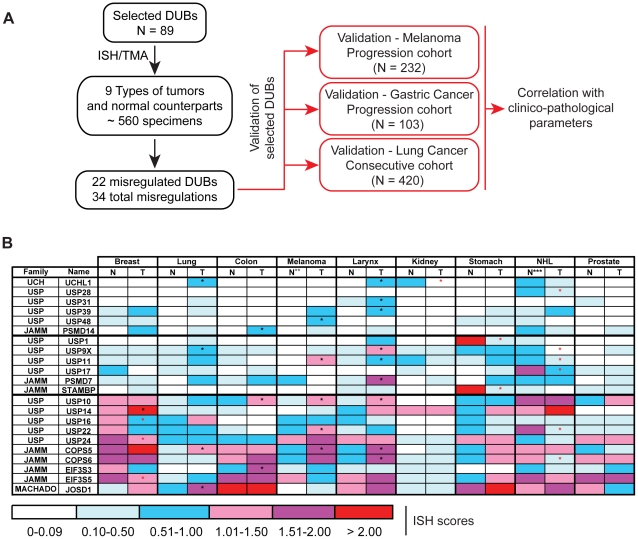
Dysregulation of DUBs in human cancers. **A**. A scheme of the study design is shown. Left (boxed in black), strategy and results of the ISH/TMA screening; right (boxed in red), strategy of the extended analyses (details are in the main text). All DUBs expressed in human tissues (90, according to [8]) were analyzed; however, the sequence of the JAMM family member ENSG00000198817 was retired from the ENSEMBL database; moreover the genomic context to which this putative transcript was assigned (chr2:58,390,463–58,391,299) (see Table S1 in [8]) now corresponds to the FANCL gene that is an E3 ligase. **B**. Dysregulation of DUBs in human cancers. The mean levels of expression in various human tumors (T) and matched normal tissues (N) are shown by a semi-quantitative color code, reflecting the mean ISH scores. Actual scores (and *P* values) are listed in Table S3. Asterisks mark statistically significant (*P*≤.05) differences (black asterisks, upregulation; red asterisks, downregulation). ** Benign nevi were used as normal tissue counterparts for melanomas. *** For non-Hodgkin's lymphomas (NHLs), we used reactive lymph node tissues as the normal counterpart.

A schematic of the study design is depicted in Figure 1A. A total of 89 DUB-encoding genes were analyzed by *in situ* hybridization (ISH) on multi-tumor tissue microarrays (TMAs). We employed ISH/TMA as the screening platform because, among RNA based technologies, ISH/TMA couples the advantages of a medium/high-throughput methodology (hundreds of genes can be screened on hundreds of tumors) with those of a high-resolution technology (each core can be analyzed by visual inspection, thereby allowing the identification of the cellular origin of the signal in a heterogeneous tissue). We have previously extensively validated the specificity and the dynamic range of detection of this method (for instance, as compared to those obtainable with highly quantitative methods, such as Q-PCR), in a number of large screening projects [23]–[26]. The ISH/TMA screening led to the identification of 22 DUBs that were dysregulated in human cancers, for a total of 34 occurrences of dysregulation (Figure 1A). Selected DUBs were subjected to further validation by analyzing their expression in large cohorts of tumor samples (Figure 1A).

### Analysis of alterations in DUB expression in human cancers by ISH/TMA

We screened by ISH/TMA ∼300 tumors, including carcinomas of the breast, colon-rectum, larynx, lung (non-small cell lung carcinomas, NSCLCs), stomach, kidney and prostate, non-Hodgkin's lymphomas (NHLs) and melanomas (the composition of the TMAs is described in Table S1). In addition, we screened ∼260 normal samples from the same tissues (frequently, and whenever possible, from the same patient, see Table S1; for NHL, we used reactive lymph node tissue as the normal counterpart, while for melanoma we used benign nevi). The 89 screened DUBs included 55 USPs, 4 UCHs, 5 MJDs, 13 OTUs, and 12 JAMMs (listed and described in Table S2).

Of the 89 analyzed transcripts, 33 (37%) could be detected (ISH score>1) in at least one of the analyzed tissues (Table S3). The remaining genes were either undetectable (40 genes) or barely (16 genes) detectable in the analyzed tissues, likely due to low mRNA abundance. Of note, in all cases in which antisense probes yielded positive signals, the corresponding sense probe, used as a negative control, did not yield any appreciable signal (data not shown). The complete set of results is shown in Table S2 and Table S3.

**Figure 2:**
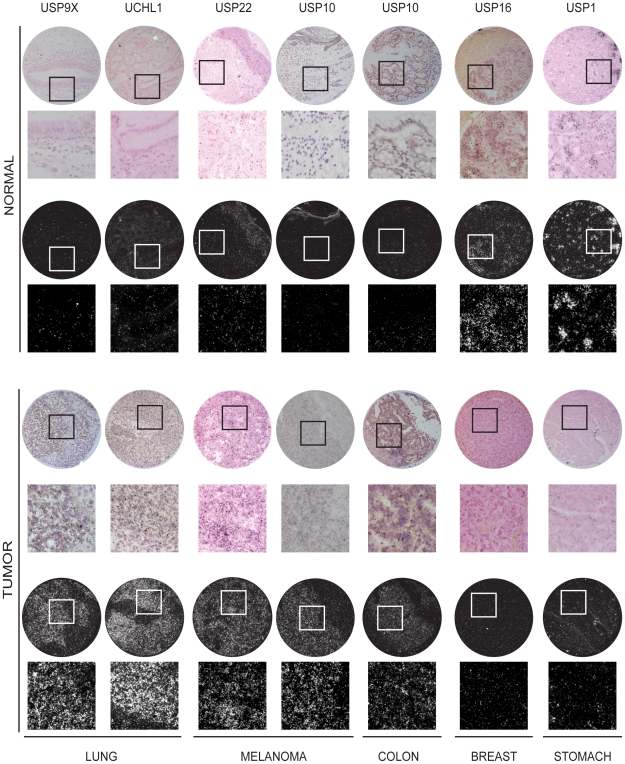
Representative examples of *in situ* hybridization-tissue microarray data. Examples of the data summarized in Figure 1B are shown for normal and tumor tissues. In each pair, the upper panel is a bright field (for morphological evaluation) and the lower panel is a dark field (transcripts appear as bright dots). Magnifications of selected areas are also shown below each individual core.

Twenty-two DUBs were dysregulated (67% of all detectable genes and ∼25% of all screened genes) in a statistically significant manner (Figure 1B, and Tables S2 and S3) in at least one tumor type (see Figure 2 for representative examples). Eleven other DUB mRNAs (CYLD, USP2, USP4, USP7, USP15, USP18, USP21, USP25, USP49, PRPF8, OTUB1) were expressed in various tissues or tumors, but were not significantly dysregulated (see Table S3). Overall, there were 34 instances in which a specific DUB was significantly dysregulated in a given tumor type, with respect to the normal counterpart; of these, 22 (65%) were upregulations, while 12 (35%) were downregulations (Figure 1B). Strikingly, 9 upregulations occurred in larynx carcinomas, while 6 downregulations occurred in NHLs. Breast carcinoma was the only tumor type in which we observed both up- and down-regulations. No DUBs were significantly dysregulated in prostate carcinomas, and only one was found in kidney (UCHL1, downregulated), suggesting that different tumor types display different levels of alteration of the deubiquitination machinery. Finally, while 15 DUBs were found to be significantly dysregulated in only one type of cancer, 7 (UCHL1, USP9X, USP11, USP10, USP22, COPS5 and COPS6) displayed multiple alterations in two or more tumor types (Figure 1B).

With therapeutic implications in mind, the most interesting DUBs were those expressed at low or undetectable levels in most normal tissues, while being upregulated in at least one tumor type. In these cases, the dysregulated DUBs frequently displayed overexpression only in a fraction of tumor samples, suggesting that their levels might also be useful for patient stratification for eligibility for anti-DUB therapy. For instance, UCHL1 in lung carcinomas (likewise in larynx carcinomas) was strongly expressed in 7 out of 25 tumor samples (28%), while it was completely undetectable in the normal counterpart. Moreover, USP31 was highly expressed in 8 out of 27 larynx carcinomas (30%), and only in 2 out of 31 normal tissues, where its expression was restricted to the proliferative basal layer (data not shown).

### Extended analysis of selected DUBs in large cohorts of tumor patients

To further investigate the alterations of DUBs in human cancers, we analyzed the expression of selected DUBs in large cohorts of human tumors. We concentrated on NSCLCs and melanomas as examples of tumors harboring frequent upregulation of DUBs, and gastric carcinomas as examples of tumors displaying downregulation of DUBs.

In NSCLCs, four DUBs were significantly overexpressed: JOSD1, COPS5 UCHL1 and USP9X (Figure 1B). JOSD1 is still totally uncharacterized at the functional level, and was therefore not further investigated. COPS5, on the other hand, has been extensively characterized in tumors, including NSCLCs (see Discussion), rendering its additional characterization less necessary. We concentrated, therefore, on the analysis of UCHL1 and USP9X by ISH/TMA on a case collection of 420 consecutive NSCLC samples (described in Table S4). We observed that UCHL1 expression directly correlated (P<0.001) with tumor grade, gender and Ki67 expression, while USP9X expression directly correlated with Ki67 expression (P<0.001; Table 1). Furthermore, both DUBs were more frequently expressed in lung squamous cell carcinomas (SSC), compared with adenocarcinomas (AC).

**Table 1:** Analysis of selected DUB expression in a large case collection of NSCLCs.

		UCHL1			USP9X		
Parameter	Group	NEG	POS	P	NEG	POS	P
**Age**	*<65*	80	59		121	23	
	*≥65*	113	81	0.911	161	48	0.137
**Nodal Status**	*Neg.*	95	68		131	44	
	*Pos.*	98	72	0.912	151	27	**0.024**
**Ki67**	*Neg.*	94	34		122	14	
	*Pos.*	78	101	**<0.001**	133	55	**<0.001**
**Grade**	*G1*	26	3		26	4	
	*G2*	84	44		114	22	
	*G3*	78	93	**<0.001**	138	44	0.133
**p53**	*Neg.*	99	58		129	32	
	*Pos.*	82	79	**0.032**	133	37	0.687
**pT**	*1–2*	156	104		217	55	
	*3–4*	37	36	0.180	65	16	1.000
**Stage**	*1*	77	52		102	35	
	*2-3-4*	116	88	0.649	180	36	0.056
**Histotype**	*AC*	123	56		170	23	
	*SCC*	70	84	**<0.001**	112	48	**<0.001**
**Gender**	*Female*	55	17		60	20	
	*Male*	138	123	**<0.001**	222	51	0.266

Correlation between DUB expression and clinico-pathological parameters in NSCLCs. Expression was measured by ISH-TMA (Negative (NEG), ISH score≤1; Positive (POS), ISH score>1). *P*-values were measured by Fisher's exact test (Pearson Chi Square was used when three or more parameters were considered). Note that the number of scored cases is lower than the total number of cases since: i) cores that gave a low β-actin signal in the control hybridization (see Methods) were excluded from further consideration; ii) in some cases, individual cores detached from the slides during the manipulations; iii) complete clinical information was not available for all patients. Histotypes: AC, adenocarcinoma; SCC, squamous cell carcinoma. In tumor tissues, the ISH signals were associated with the tumor cell component and not with the adjacent or infiltrating stroma.

In the initial screening of melanoma samples, five genes (USP10, USP11, USP22, USP48 and COPS5) were significantly overexpressed, compared with benign nevi. We performed an in-depth analysis on four of them (COPS5 was not analyzed for the reasons mentioned in the previous paragraph) on a large melanoma case collection (described in Table S5). The expression of three out of four analyzed genes (USP10, USP11, USP22) was significantly higher in metastatic melanoma compared with benign nevi and primitive tumors (Table 2), suggesting that their expression is associated with a more aggressive and invasive phenotype. This conclusion is supported by the significant correlation observed between DUB expression and clinico-pathological parameters indicative of advanced disease (Table 2), including the Breslow index (for USP10 and USP22), the Clark index (for USP22, USP11 displayed a borderline correlation), the presence of ulceration (for USP10 and USP22), and the number of mitotic cells (for USP10 and USP22, USP11 displayed a borderline correlation). USP48 expression did not correlate with any clinico-pathological parameters since low levels of transcript were detected in almost all tumor samples (>95%, data not shown). Thus, in a significant number of melanoma cases, DUB expression correlated with some of the strongest known prognostic factors, projecting their usefulness in prognostic models.

**Table 2:** Analysis of selected DUB expression in melanoma progression.

		USP10			USP11			USP22		
Parameter	Group	NEG	POS	P	NEG	POS	P	NEG	POS	P
**Type**	*Nevi*	20	0		25	0		21	0	
	*Melanoma*	104	8		121	10		99	25	
	*Met. Mel.*	34	19	**<0.001**	35	18	**<0.001**	27	25	**<0.001**
**Histotype**	*NM*	13	2		14	1		9	6	
	*SSM*	90	5	0.243	105	7	0.950	89	16	**0.031**
**pT**	*1*	21	0		28	0		22	0	
	*2–4*	47	8	0.097	52	7	0.091	38	22	**<0.001**
**Nodal status**	*Neg.*	23	5		32	4		17	17	
	*Pos.*	8	3	0.663	9	2	0.614	8	3	0.297
**Regression**	*No*	74	4		85	5		67	20	
	*Yes*	29	3	0.413	34	3	0.691	30	3	0.119
**Ulceration**	*No*	75	1		88	5		77	8	
	*Yes*	28	6	**0.003**	32	3	0.683	21	15	**<0.001**
**TIL**	*No*	67	3		77	4		64	12	
	*Yes*	36	5	0.143	42	5	0.287	33	12	0.163
**Mitotic Count**	*0–1*	42	0		49	0		44	2	
	*2–6*	36	2		37	4		28	10	
	*>6*	17	6	**<0.001**	20	4	**0.022**	13	11	**<0.001**
**Breslow**	*0–1*	56	0		67	2		62	0	
	*2–3*	29	2		31	4		23	11	
	*3+*	18	6	**<0.001**	22	3	0.149	13	13	**<0.001**
**Clark**	*1–2*	30	0		42	0		37	0	
	*3–5*	72	8	0.104	77	9	**0.029**	60	24	**<0.001**
**Age**	*<65*	75	26		83	35		69	29	
	*≥65*	5	3	0.436	8	1	0.444	14	7	0.795
**Gender**	*Female*	42	4		50	4		43	10	
	*Male*	62	4	0.715	71	5	0.855	56	14	0.875

Correlation between DUB expression and clinico-pathological parameters in melanomas. Expression was measured by ISH-TMA (Negative (NEG), ISH score≤1; Positive (POS), ISH score>1). *P*-values were measured as in Table 1. Note that the number of scored cases is lower than the total number of cases (see Table 1). Histotypes: NM, nodular melanoma; SSM, superficial spreading melanoma. TIL: tumor-infiltrating lymphocytes. In all melanomas (including metastatic ones), the ISH signals were associated with the tumor cell component and not with the adjacent or infiltrating stroma.

Finally, we measured the expression of USP1 on a gastric cancer “progression” TMA containing normal gastric epithelia, intestinal metaplasia, dysplasia, primary carcinomas and metastases (Table S6). We observed that the expression of USP1 was lost in the transition from the normal to the metaplastic state (Table 3, see also Figure 1B and Figure S1). All abnormal and neoplastic gastric tissues were negative for USP1 expression, possibly indicating that this event correlates with the initial steps of transformation of the gastric mucosa.

**Table 3:** Analysis of USP1 expression in the progression of gastric cancer.

	Analyzed Samples	Positive	Negative	% Positive
**Normal Mucosa**	18	17	1	92
**Intestinal Metaplasia**	14	0	14	0
**Displasia**	9	0	9	0
**Primary tumor**	28	0	28	0
**Metastasis**	13	0	13	0

USP1 expression was measured in normal, metaplastic, dysplastic and neoplastic gastric tissues by ISH-TMA (Negative, ISH score≤1; Positive, ISH score>1). In the normal gastric mucosa the ISH signals were observed throughout the thickness of the epithelial component, irrespectively to the type of glands analyzed.

## Discussion

Herein, we provide the first atlas of alterations of DUB expression in human cancers. The complete repertoire of DUBs encoded by the human genome was analyzed in nine types of cancer, which included the four most frequent cancers (lung, prostate, breast, colon-rectum), and which account for ∼two thirds of all cancer cases and cancer deaths in the western world.

Twenty-two DUBs were found to be significantly dysregulated in at least one type of cancer. In seven cases (UCHL1, USP9X, USP11, USP10, USP22, COPS5 and COPS6), dysregulation was observed in more than one tumor type. Considering that only 33 of the 89 screened DUBs displayed quantifiable ISH signals, it appears that these enzymes are frequently altered in human cancers. Obviously, dysregulation in tumors does not constitute *per se* evidence for a causal involvement in cancer. In our extended analyses, however, we observed an association between the expression of selected DUBs and relevant clinico-pathalogical parameters, in some cases indicative of aggressive disease. These data support the notion that at least some of the detected dysregulations might have a role in tumorigenesis. In addition, some of the characterized DUBs might provide useful markers for diagnostic/prognostic evaluation (e.g., USP10, USP11 and USP22 in melanoma), or might represent therapeutic targets (e.g., DUBs that are highly expressed in tumors, but absent in normal tissues), regardless of their exact role in tumorigenesis.

Several of the dysregulated DUBs identified here have already been shown to be involved in cancer (for recent reviews see [18], [19]). For instance, COPS5 is overexpressed in several tumor types [27], [28], and its overexpression is associated with short disease-free and overall survival in lung cancer [29], [30]. Indeed, COPS5 has been proposed as a target for anti-cancer drug development [31].

USP9X expression has been shown to promote the self-renewal of embryonic stem cell-derived neural progenitors, acting as a neural stemness gene [32]; it promotes cell survival by stabilizing MCL1, which is essential for the survival of stem and progenitor cells of multiple lineages [33]. We found USP9X overexpressed in lung cancer, suggesting that this event might be linked to the expansion of the cancer stem cell compartment in this tumor: a possibility that warrants further investigation.

Upregulation of UCHL1 expression was observed in bronchial biopsies of smokers compared with non-smokers [34], and its expression has been linked to disease outcome in lung cancer [35], [36]. Moreover UCHL1 expression in cancer-associated fibroblasts of colorectal cancer was found to be an independent prognostic factor for overall and recurrence-free survival [37]. Finally, its overexpression strongly accelerated lymphomagenesis in Eμ-myc transgenic mice through the enhancement of AKT signaling [38].

Another example is represented by USP22, which is part of a small set of marker genes capable of predicting metastatic potential and therapeutic outcome in human cancer [39], [40]. USP22 is overexpressed in colorectal cancer and its activation is associated with tumor progression and therapy failure [41]. USP22 may exert its oncogenic potential through the BMI-1 oncogene-driven pathway signature by activating c-Myc-targeted genes, such as cyclin D2 [41]. Notably treatment with USP22-specific siRNA and aiRNA (asymmetric interfering RNA) inhibits the growth of implanted bladder tumors in vivo [42], possibly through the downregulation of Mdm2 and cyclin E, resulting in the stabilization of p53 and p21 and ensuing cell cycle arrest [42].

In all these cases, our findings support the notion that these DUBs play an important role in human cancer, and further pose the question of which are the molecular mechanisms responsible for their dysregulation. In addition, it will be of interest to test whether genetic alterations directly affecting the genes for theses enzymes can be evidenced in cancer.

Conversely, for many other DUBs (USP31, USP39, USP48, PSMD14, USP1, PSMD7, STAMBP, USP16, USP24, COPS6, EIF3S5 and JOSD1) our findings represent, to the best of our knowledge, the first report of alterations in cancer. Two of these DUBs, PSMD7 and PSDM14, are component of the proteasome, and might therefore be of direct relevance to therapy, including patient stratification, in light of the fact that the proteasome inhibitor bortezomib has already been approved for the treatment of multiple myeloma and mantle cell lymphoma [43], and that additional clinical trials for the treatment of solid tumors and other hematological malignancies are in progress [44]. In particular, PSMD14 was identified as an important DUB of the 19S lid complex of the proteasome [45]. Its activity is essential for substrate deubiquitination during proteasomal degradation [46], and may also play a role in the editing of polyubiquitinated substrates as a mean to control degradation, possibly in a proteasomal-independent fashion [47], [48]. Moreover PSMD14 has been shown to deubiquitinate the transcription factor JUN; its overexpression contributes to JUN stabilization and activation of its downstream target genes [49], thereby conferring moderate resistance to chemotherapeutic drugs [50]. It will be therefore of interest to evaluate whether the possible contribution of PSMD14 to human cancer occurs through proteosomal-dependent or –independent functions.

The possible relevance of DUB dysregulation to human cancers is best appreciated in the framework of available knowledge on their role in biochemical circuitries involved in cellular regulation. While a comprehensive discussion of the known functions of all the dysregulated DUBs identified in this study will be impossible here (see however Table S2 and [8], [18], [19] for recent reviews of the biochemical functions of DUBs implicated in cancer), we would like to briefly highlight some of the functional characteristics of the DUBs that were extensively validated in the present study (USP9X, UCHL1, USP1, USP10, USP11, and USP22). These DUBs are involved in the regulation of cellular functions relevant to cancer, including signal transduction pathways, apoptosis, transcription, regulation of chromatin, and DNA repair processes.

USP9X, UCHL1 and USP11 have been implicated in the regulation of signal transduction pathways. USP9X interacts with β-catenin *in vitro* and *in vivo* [51], [52] and probably mediates its deubiquitination, thereby increasing its half-life [51]. UCHL1 might be involved in the same pathway, since it forms endogenous complexes with β-catenin, stabilizes it, and upregulates β-catenin/TCF-dependent transcription [53]. Moreover, UCHL1 and β-catenin can positively regulate each other [53]. The effects of USP9X, and possibly of UCHL1, might therefore mimic activation of the Wnt signaling pathway, which is known to cause β-catenin stabilization and translocation into the nucleus, and has been implicated in a variety of human cancers (for reviews see [54]–[57]). USP9X might also act as a regulator of the TGF-β pathway, another signaling circuitry of great relevance to cancer (reviewed in [58]), as witnessed by the fact that loss of USP9X abolishes multiple TGF-β gene responses [59]. Mechanistically, this might depend on the ability of USP9X to activate SMAD4 by removing its monoubiquitination, which in turn prevents the formation of the effector SMAD2/SMAD4 complex [59]. Finally, USP11 is involved in the regulation of the NF-κB signaling pathway [60], [61].

There is evidence that USP9X and USP10 might be involved in cell survival pathways. USP9X deubiquitinates and stabilizes MCL-1, a pro-survival BCL2 family member [33], whose overexpression is associated with several neoplastic conditions [62]–[64]. USP10, on the other hand, has been shown to be responsible for the deubiquitination of p53 in the cytoplasm, allowing its stabilization and re-entry into the nucleus. Indeed, downregulation of USP10 decreases p53 stability and increases cancer cell proliferation [65], thus projecting a role as a tumor suppressor. Interestingly, however, USP10 can also act like an oncogene, by promoting cancer cell proliferation in cells harboring mutant p53 [65], an event possibly connected with the fact that some p53 mutants display aberrant gain-of-function activity that is stabilized through deubiquitination by USP10.

There is also evidence for an involvement of USP22 and USP1 in a series of nuclear events, including organization of chromatin and telomeres, and DNA repair, the subversion of which might lead to cellular transformation. USP22 is necessary for appropriate progression through the cell cycle, and it is a component of the human SAGA complex, a transcriptional co-activator complex. Within SAGA, USP22 catalyzes the deubiquitination of histones 2A and 2B, thereby, counteracting heterochromatin silencing [66]. Moreover, it deubiquitinates TRF1, a component of the telomere nucleoprotein complex that functions as an inhibitor of telomerase [67], thereby affecting TRF1 stability and telomere elongation [68]. Finally, USP1 deubiquitinates and inactivates two components of DNA repair mechanisms: FANCD2 (a component of the Fanconi Anemia pathway) [4], [69] and PCNA [70]. Ubiquitination of FANCD2 and PCNA is important for their roles in DNA repair [71], [72], suggesting that subversion of USP1 in human cancers might impinge on transformation events through alterations of DNA repair pathways.

Finally, the interactome of human DUBs has been recently reported [73], which links DUBs to diverse cellular processes, including protein turnover, transcription, RNA processing, DNA damage, and endoplasmic reticulum-associated degradation. The DUB interactome provides the foundations, onto which additional layers of complexity can now be added, such as the atlas of DUB alterations in cancer reported herein, to build a reference map for the pleiotropic involvement of DUBs in cellular homeostasis.

## Materials and Methods

### Ethics statement

Written informed consent for research use of biological samples was obtained from all patients, and the research project was approved by the Institutional Ethical Committee. Current Members of the IEO Ethics Committee: Luciano Martini (Chairman), Director of the Institute of Endocrinology, Milan; Apolone Giovanni (Vice Chairman), Chief of the Translational and Outcome Research Laboratory and the “Mario Negri” Institute, Milan; Bonardi Maria Santina, Head of the Nursing Service – European Institute of Oncology, Milan; Cascinelli Natale, Scientific Director – National Cancer Institute, Milan; Gallus Giuseppe, Director – Institute of Medical Statistics – Milan; Gastaldi Stefano, Psychologist and Psychotherapist, Scientific Director of Attivecomeprima; Goldhirsch Aron, Director of the Department of Medicine – European Institute of Oncology, Milan; La Pietra Leonardo, Chief Medical Officer – European Institute of Oncology, Milan; Loi Umberto, Export in Legal Procedures, Monza; Martini Luciano (Presidente), Director - Institute of Endocrinology, Milan; Merzagora Francesca, President of Italian Forum of Europa Donna, Milan; Omodeo Sale' Emanuela, Director of Pharmaceutical Service, European Institute of Oncology, Milan; Pellegrini Maurizio, Head of the Local Health District, Milan; Rotmensz Nicole, Head of the Quality Control Unit, European Institute of Oncology, Milan; Tomamichel Michele, Director, Sottoceneri Sector Cantonal Sociopsychiatric Organisation, Lugano; Monsignor Vella Charles, Bioethicist and theologist, S. Raffaele Hospital and Scientific Institute, Milan; Veronesi Umberto, Scientific Director, European Institute of Oncology, Milan.

OBSERVERS: Ciani Carlo, Chief Executive Officer, European Institute of Oncology, Milan; Della Porta Giuseppe, Research Co-ordinator, European Institute of Oncology, Milan; Michelini Stefano, Managing Director, European Institute of Oncology, Milan.

SECRETERIAT OFFICE: Nonis Atanasio (head), Controlled Clinical Studies Office, European Institute of Oncology, Milan; Tamagni Daniela (Assistant), Controlled Clinical Studies Office, European Institute of Oncology, Milan.

### Identification and selection of DUBs, cDNA templates and probe preparation

We used the Pfam (Pfam 22.0, July 2007) [74], the InterPro (InterPro 16.0, August 2007, http://www.ebi.ac.uk/interpro/), and the SMART databases to retrieve all proteins containing one of the five Ub-protease domains. After removing overlapping sequences, we identified 55 USPs, 4 UCHs, 5 MJDs, 13 OTUs, and 12 JAMMs, which represented the 89 genes screened on TMAs (Table S2).

EST clones were obtained from our in-house Unigene clone collection, or from IMAGENES (http://www.imagenes.de/). All clones were sequence verified. BLAST searches were performed to identify the most specific ∼300 bp regions, shared by the highest number of transcript variants for each individual gene, and riboprobes were synthesized as described previously [23].

### Tissue samples

For the large-scale screening study, formalin fixed and paraffin embedded specimens were provided by the Pathology Departments of Ospedale Maggiore (Novara), Presidio Ospedaliero (Vimercate), and Ospedale Sacco (Milan). Samples were arrayed onto different TMAs (Table S1), prepared essentially as previously described [23], [25], [75]. Each sample was arrayed in duplicate (also for the TMAs engineered for the extended analyses, see below). Details of the TMA engineering are in Table S1.

For the extended analyses of representative DUBs, we used three different cohorts:

1. Lung cancer cohort. We designed lung-specific TMAs composed of 420 NSCLCs (244 adenocarcinomas and 176 squamous cell carcinomas), provided by the European Institute of Oncology (Milan). Clinical and pathological characteristics are reported in Table S4.
2. Melanoma cohort. We designed a melanoma-progression TMA composed of 32 benign lesions (nevi), 138 primary melanomas, and 62 metastatic melanomas provided by the Pathology Departments of Ospedale S. Paolo (Milan) and by the European Institute of Oncology (Milan). Clinical and pathological characteristics are in Table S5.
3. Gastric cancer cohort. This cohort was arrayed onto a gastric cancer progression TMA, which contained 31 primary gastric carcinomas (8 early and 23 advanced) provided by Ospedale S. Paolo (Milan) and Fondazione IRCCS Ospedale Maggiore Policlinico, Mangiagalli e Regina Elena (Milan). Non-neoplastic specimens (13 dysplasias, 23 intestinal metaplasias and 23 normal mucosae) and 13 lymph node metastases from the same patients were also arrayed. Clinical and pathological characteristics are reported in Table S6.

### *In situ* hybridization (ISH)

ISH was performed as previously described [23]. All TMAs were first analyzed for the expression of the housekeeping gene β-actin, to check the mRNA quality of the samples. Cases showing absent or low β-actin signals were excluded from the analyses (data not shown). In addition, in all cases where antisense probes yielded positive signals (33 genes), the corresponding sense probe, used as a negative control, did not yield any appreciable signal. Gene expression levels were evaluated by counting the number of grains per cell and were expressed on a semi-quantitative scale (ISH score): 0 (no staining), 1 (1–25 grains; weak staining), 2 (26–50 grains, moderate staining), and 3 (>50 grains, strong).

The mean ISH score was calculated when two (or more) cores of the same sample were present. Mean ISH scores>1 were considered as an unequivocal positive signal. Mean ISH scores between 0 and 1 were considered as a negative signal. Note that the number of scored cases, in some experiments, is lower than the total number of arrayed cases. This is due to a number of reasons: i) all cores that gave a low β-actin signal in the control hybridization were excluded from further consideration; ii) in some cases, individual cores detached from the slide during the manipulations.

### Statistical analysis

Dysregulation (up- or down-regulation) was evaluated by assessing differences between the tumor and the normal groups with the Fisher's exact test and the Student's t-test, and only if the difference in the average scores between the two groups was >0.5. Differences were judged as significant at confidence levels equal to or greater than 95% (*p*≤0.05; see Table S3). Analyses were performed using JMP statistical software (SAS Institute, Inc., Cary, NC). The association between clinico-pathological parameters of the tumors and DUB expression in the melanoma and lung tumor cohorts was evaluated using the Fisher's exact test, or with the Pearson chi-square test when three or more parameters were evaluated at the same time.

## Supporting Information

Figure S1 **High resolution images of data presented in** **Figure 2** **of the main text.** High magnifications of the TMA core showing USP1 expression in normal gastric mucosa. Top, hematoxylin/eosin staining; bottom, dark field. The boxed areas highlight the presence of a region of intestinal metaplasia, within the normal gastric mucosa, showing the absence of USP1.
(TIF)

Table S1 **Four different multi-tissue TMAs (indicated as TMA A–D) were prepared.** For their engineering, two tumor areas (diameter 0.6 mm) and two normal areas (0.6 mm, arrayed whenever available) from each specimen were first identified on haematoxylin-eosin stained sections, and subsequently removed from the donor blocks and deposited on the recipient block using a custom-built precision instrument (Tissue Arrayer-Beecher Instruments, Sun Prairie, WI 53590, USA) coupled to a motorization kit (MTABooster-Alphelys, Plaisir, France). We also engineered two TMAs containing only normal tissues (normal TMAs 1 and 2), from different donors. In this case, we deposited in duplicate larger cores (diameter 1.5 mm) to allow for better characterization of the normal tissue architecture. In each column, the number of cases deposited on each TMA is reported (T, tumor; N, normal). * For non-Hodgkin's lymphoma (NHL), we used reactive lymph node tissues as the normal counterpart. ** For melanomas, benign nevi were used as the normal tissue counterpart. Different TMA combinations were used for the screening. With reference to the 33 detectable genes: USP49 and OTUB1 were hybridized only to TMA D and Normal TMA-2; EIF3S5, PRPF8 and PSMD7 to TMAs C–D and to Normal TMAs 1–2; STAMBP, COPS6 and EIF3S3 to TMAs A–D and to Normal TMAs 1–2; all other 25 genes were hybridized on TMAs A–C and to Normal TMA-1.
(DOC)

Table S2 **List of screened DUBs identified by their family name (UCH, USP, OTUBIAN, MACADO, JAMM), symbol (HUGO nomenclature, used throughout this paper), definition, aliases (if known), accession numbers (mRNA Acc ID, Prot Acc ID, and EST Acc/RZPD clones ID), function (see below), and relevant references.** The column “TMA Expression” reports delectability in the ISH/TMA procedure. Functions were derived by merging information obtained from PubMed and the GeneCards Database (http://bioinfo1.weizmann.ac.il/genecards/index.shtml). n.a.: not available.
(DOC)

Table S3 **Mean values of the data presented in** **Figure 1B** **of the main text.** The mean ISH score of replicate cores arrayed in the TMAs was used to calculate the final ISH scores for each tumor sample. Number of normal and tumor samples analyzed (n°N and n°T respectively) and number of positive samples are reported for each screened tissue. Mean gene expression was calculated as indicated in the Methods and is reported separately for tumor (Average T) and normal tissues (Average N). Statistical significance between mean expression levels in T and N was calculated as reported in the Methods and P-values are reported. In tumor tissues, in all cases, the ISH signals of the over- or under-expressed genes were associated with the tumor cell component and not with the adjacent or infiltrating stroma. The same was true for normal tissues.
(XLS)

Table S4 **The clinical and pathological information for the patients of the NSCLC cohort is reported.** For some patients not all information was available (No data). *For three patients no follow-up was available.
(DOC)

Table S5 **The clinical and pathological information for the patients of the melanoma cohort is shown.** Clinical parameters are reported only for the 138 primary melanomas. For some patients not all information was available (No data). Histotypes: NM, Nodular Melanoma; SSM, Superficial Spreading Melanoma; TIL: Tumor-Infiltrating Lymphocytes.
(DOC)

Table S6 **The clinical and pathological information for the patients of the gastric cancer cohort is reported.** For some patients not all information was available (No data). Class: EGC, early gastric cancer; AGC, advanced gastric cancer.
(DOC)
