# Supplementary figures and images for: An Atlas of Altered Expression of Deubiquitinating Enzymes in Human Cancer
Source: PLoS One. 2011 Jan 25;6(1):e15891. doi: 10.1371/journal.pone.0015891 (PMC3026797; doi:10.1371/journal.pone.0015891)

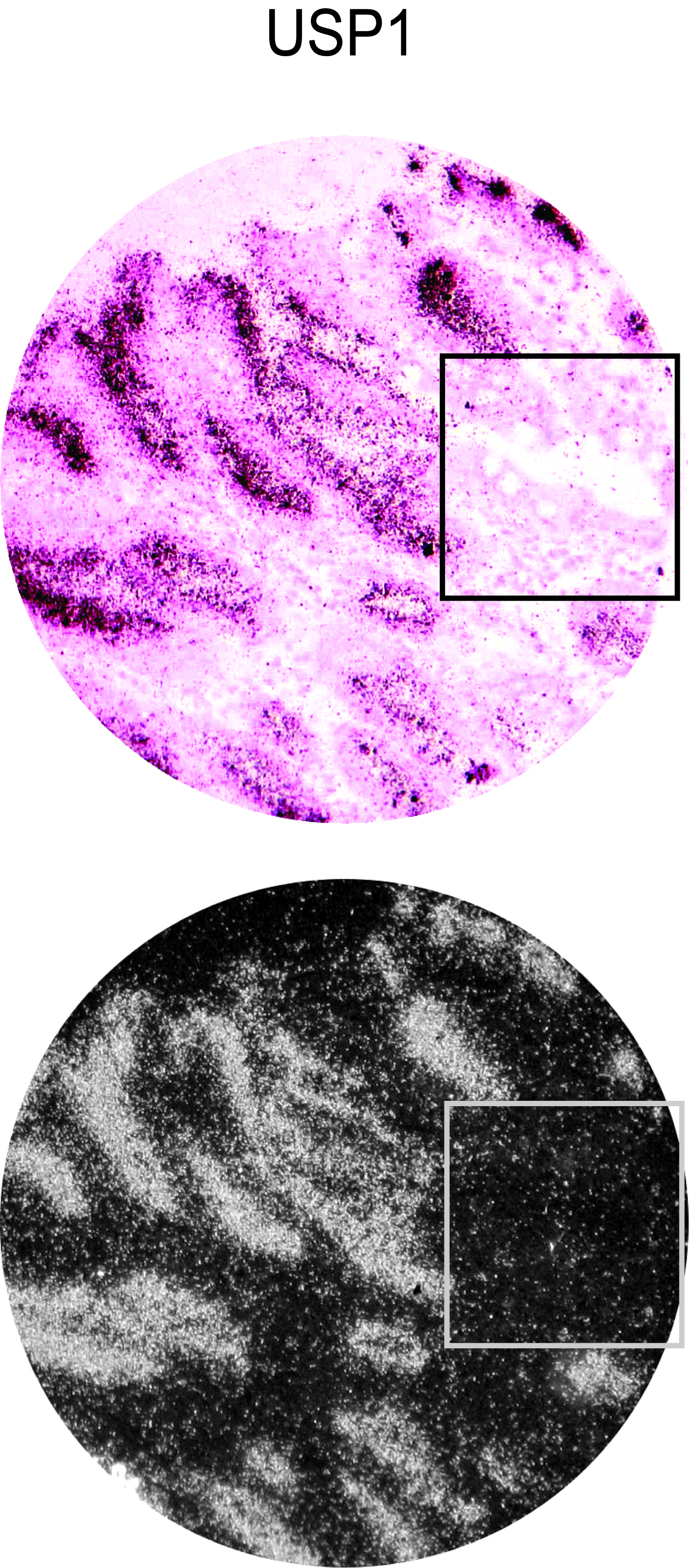

Supplement: Figure S1 — High resolution images of data presented in Figure 2 of the main text. High magnifications of the TMA core showing USP1 expression in normal gastric mucosa. Top, hematoxylin/eosin staining; bottom, dark field. The boxed areas highlight the presence of a region of intestinal metaplasia, within the normal gastric mucosa, showing the absence of USP1. (TIF) [file pone.0015891.s001.tif]
