# Supplementary material for: An Atlas of Altered Expression of Deubiquitinating Enzymes in Human Cancer
Source: PLoS One. 2011 Jan 25;6(1):e15891. doi: 10.1371/journal.pone.0015891 (PMC3026797; doi:10.1371/journal.pone.0015891)
Supplement: Table S1 — Four different multi-tissue TMAs (indicated as TMA A–D) were prepared. For their engineering, two tumor areas (diameter 0.6 mm) and two normal areas (0.6 mm, arrayed whenever available) from each specimen were first identified on haematoxylin-eosin stained sections, and subsequently removed from the donor blocks and deposited on the recipient block using a custom-built precision instrument (Tissue Arrayer-Beecher Instruments, Sun Prairie, WI 53590, USA) coupled to a motorization kit (MTABooster-Alphelys, Plaisir, France). We also engineered two TMAs containing only normal tissues (normal TMAs 1 and 2), from different donors. In this case, we deposited in duplicate larger cores (diameter 1.5 mm) to allow for better characterization of the normal tissue architecture. In each column, the number of cases deposited on each TMA is reported (T, tumor; N, normal). * For non-Hodgkin's lymphoma (NHL), we used reactive lymph node tissues as the normal counterpart. ** For melanomas, benign nevi were used as the normal tissue counterpart. Different TMA combinations were used for the screening. With reference to the 33 detectable genes: USP49 and OTUB1 were hybridized only to TMA D and Normal TMA-2; EIF3S5, PRPF8 and PSMD7 to TMAs C–D and to Normal TMAs 1–2; STAMBP, COPS6 and EIF3S3 to TMAs A–D and to Normal TMAs 1–2; all other 25 genes were hybridized on TMAs A–C and to Normal TMA-1. (DOC) [file pone.0015891.s002.doc]

**Table S1. Engineering of the TMAs used for the initial screening**

|  | **Breast** | | **Colon** | | **Larynx** | | **Lung** | | **NHL** | | **Stomach** | | **Kidney** | | **Prostate** | | **Melanoma** | |
| --- | --- | --- | --- | --- | --- | --- | --- | --- | --- | --- | --- | --- | --- | --- | --- | --- | --- | --- |
|  | **T** | **N** | **T** | **N** | **T** | **N** | **T** | **N** | **T** | **N*** | **T** | **N** | **T** | **N** | **T** | **N** | **T** | **N**** |
| **TMA A** |  |  |  |  | 28 | 28 | 28 | 28 |  |  | 20 | 20 | 20 | 20 |  |  |  |  |
| **TMA B** | 24 | 14 |  |  | 1 |  |  |  | 29 | 6 |  |  |  |  |  |  | 16 | 13 |
| **TMA C** |  |  | 28 | 28 |  |  |  |  |  |  |  |  |  |  | 20 | 20 |  |  |
| **TMA D** | 33 | 2 | 34 | 2 | 32 | 2 | 33 | 2 | 30 | 2 | 34 | 2 | 28 | 2 | 25 | 2 | 15 | 11 |
| **Normal TMA-1** |  | 9 |  | 10 |  | 10 |  | 10 |  | 5 |  | 9 |  |  |  |  |  |  |
| **Normal TMA-2** |  | 13 |  | 8 | 1 | 9 |  | 11 |  | 7 |  | 10 |  | 10 |  | 7 |  |  |
| **TOTAL** | 57 | 38 | 62 | 48 | 62 | 49 | 61 | 51 | 59 | 20 | 54 | 41 | 48 | 32 | 45 | 29 | 31 | 24 |
| **TOTAL without common samples** | 33 | 37 | 36 | 39 | 39 | 40 | 38 | 40 | 36 | 11 | 35 | 30 | 37 | 30 | 25 | 24 | 16 | 13 |
