# Supplementary material for: An Atlas of Altered Expression of Deubiquitinating Enzymes in Human Cancer
Source: PLoS One. 2011 Jan 25;6(1):e15891. doi: 10.1371/journal.pone.0015891 (PMC3026797; doi:10.1371/journal.pone.0015891)
Supplement: Table S2 — List of screened DUBs identified by their family name (UCH, USP, OTUBIAN, MACADO, JAMM), symbol (HUGO nomenclature, used throughout this paper), definition, aliases (if known), accession numbers (mRNA Acc ID, Prot Acc ID, and EST Acc/RZPD clones ID), function (see below), and relevant references. The column “TMA Expression” reports delectability in the ISH/TMA procedure. Functions were derived by merging information obtained from PubMed and the GeneCards Database (http://bioinfo1.weizmann.ac.il/genecards/index.shtml). n.a.: not available. (DOC) [file pone.0015891.s003.doc]

**Table S2. List of screened Deubiquitinating Enzymes (DUBs).**

| **FAMILY** | **SYMBOL** | **NAME** | **ALIASES** | **mRNA Acc ID** | **Prot Acc ID** | **EST Acc/RZPD clones ID** | **FUNCTION** | **Ref** | **TMA Expression** |
| --- | --- | --- | --- | --- | --- | --- | --- | --- | --- |
| **UCH** | BAP1 | BRCA1 associated protein-1 (ubiquitin carboxy-terminal hydrolase) | hucep-6, HUCEP-13, KIAA0272 | NM_004656 | NP_004647 | H09065 | BAP1 is a candidate tumor suppressor gene that regulates cell cycle progression. It acts in a BRCA1-dependent and -independent fashion to inhibit cell proliferation. BAP1 also forms complexes with transcription factors and cofactors and regulates the expression of genes regulating proliferation. | [1,2] | **Undetectable** |
| **UCH** | UCHL1 | ubiquitin carboxyl-terminal esterase L1 (ubiquitin thiolesterase) | PARK5, PGP9.5 | NM_004181 | NP_004172 | AA670438 | UCHL1 is believed to be important for neural cell function and male fertility. It has been shown to be involved in the regulation of TP53 activity and apoptosis. Its dysregulation has been linked to neurodegenerative disorders and cancer, where it has been described as both a tumor suppressor and an oncogene. | [3,4] | **Detectable** |
| **UCH** | UCHL3 | ubiquitin carboxyl-terminal esterase L3 (ubiquitin thiolesterase) | UCH-L3 | NM_006002 | NP_005993 | IMAGp958P082638Q | UCHL3 is also a deneddylating enzmye. Its functions are still unclear, although UCHL3 knockout mice display retinal and skeletal muscle degeneration, cryptorchid injury, resistance to obesity, accumulation of polyubiquitinated proteins, and activation of the cellular stress response. It is also involved in endocytic recycling of ion channels. | [5] | **Barely detectable** |
| **UCH** | UCHL5 | ubiquitin carboxyl-terminal hydrolase L5 | UCH37, CGI-70, INO80R | NM_015984 | NP_057068 | IRATp970E0318D | UCHL5 is associated with the proteasome and the Ino80 chromatin-remodeling complex. It has also been shown to interact with Smads and regulate TGF-beta signaling. | [6,7] | **Undetectable** |
| **USP** | CYLD | cylindromatosis (turban tumor syndrome) | EAC, CDMT, CYLD1, HSPC057, KIAA0849 | NM_015247 | NP_056062 | R62469 | CYLD regulates diverse physiological processes, such as the inflammation, cell cycle progression, spermatogenesis, and osteoclastogenesis. It negatively regulates several signaling pathways, including NF-kappaB and Wnt/β-catenin signaling. CYLD is also is a tumor suppressor and mutations in this gene are associated with tumor predisposition syndromes, e.g., cylindromatosis, multiple familial trichoepithelioma, and Brooke-Spiegler syndrome. | [8,9] | **Detectable** |
| **USP** | PAN2 | PAN2 poly(A) specific ribonuclease subunit homolog (S. cerevisiae) | USP52, hPAN2, FLJ39360, KIAA0710 | NM_014871 | NP_055686 | AA489635 | PAN2 complexed with hPan3 is a major poly(A) nuclease involved in cytoplasmic mRNA decay. PAN2 does not display DUB activity *in vitro.* | [10] | **Undetectable** |
| **USP** | USP1 | ubiquitin specific protease 1 | UBP | NM_003368 | NP_003359 | DKFZp434A028Q | USP1 binds to, and is activated by, UAF1. USP1 regulates the Fanconi anemia, and the PCNA-mediated, DNA repair pathways. It is also involved in hematopoietic stem cell. | [11,12] | **Detectable** |
| **USP** | USP2 | ubiquitin specific peptidase 2 | USP9, UBP41 | NM_004205 | NP_004196 | AI215004 | USP2 regulates the p53 pathway, by deubiquitinating, thereby stabilizing, Mdm2, which in turn promotes p53 degradation. USP2 may also involved in the regulation of cell cycle progression and apoptosis. | [13,14] | **Detectable** |
| **USP** | USP3 | ubiquitin specific protease 3 | UBP, SIH003, MGC129878, MGC129879 | NM_006537 | NP_006528 | IRATp970B0319D | USP3 is a chromatin modifier required for S phase progression and genome stability. | [15] | **Undetectable** |
| **USP** | USP4 | ubiquitin specific peptidase 4 (proto-oncogene) | UNP, Unph, MGC149848, MGC149849 | NM_003363 | NP_003354 | AA454143 | USP4 is an oncogenic protein that interacts with the Rb family of tumor suppressor proteins. | [16,17] | **Detectable** |
| **USP** | USP5 | ubiquitin specific peptidase 5 (isopeptidase T) | ISOT | NM_001098536 | NP_001092006 | AA465536 | USP5 deubiquitinates unanchored polyubiquitin. It also regulates p53 activity; suppression of USP5 causes an accumulation of unanchored polyubiquitin, which interferes with proteosomal degradation of ubiquitinated p53. | [18] | **Undetectable** |
| **USP** | USP6 | ubiquitin specific peptidase 6 (Tre-2 oncogene) | HRP1, TRE2, TRE17, Tre-2 | NM_004505 | NP_004496 | IRATp970G0532D | USP6 is an oncogene that is expressed in a variety of human cancers. It is an effector of Cdc42 and Rac1 in actin remodeling and an activator of ARF6 in the plasma membrane recycling system. | [19-21] | **Barely detectable** |
| **USP** | USP7 | ubiquitin specific peptidase 7 (herpes virus-associated) | TEF1, HAUSP | NM_003470 | NP_003461 | IMAGp958P131848Q | USP7 is a critical regulator of p53 that deubiquitinates both p53 and Mdm2. It also modulates transcriptional repression through the Polycomb repressive complex 1. | [22-24] | **Detectable** |
| **USP** | USP8 | ubiquitin specific peptidase 8 | UBPY, HumORF8, FLJ34456, KIAA0055, MGC129718 | NM_005154 | NP_005145 | AI299198 | USP8 is an essential growth-regulated enzyme required for cell proliferation and survival. It regulates endosomal sorting and receptor trafficking. It has also been detected as an oncogenic fusion protein in CML. | [25,26] | **Barely detectable** |
| **USP** | USP9X | ubiquitin specific peptidase 9, X-linked | FAF, FAM, DFFRX | NM_001039590 | NP_001034679 | R93207 | USP9x is required for the formation of cell-cell junctions during epithelial cell polarization. It also promotes cell survival by stabilizing MCL-1, and is essential for TGFbeta and bone morphogenetic protein signaling. | [27,28] | **Detectable** |
| **USP** | USP9Y | ubiquitin specific protease 9, Y-linked | DFFRY, FLJ33043 | NM_004654 | NP_004645 | HU3_p983A01260D | USP9Y has been linked to spermatogenesis, however, recent evidence suggests that it is not essential for this process. | [29,30] | **Undetectable** |
| **USP** | USP10 | ubiquitin specific protease 10 | UBPO, KIAA0190 | NM_005153 | NP_005144 | AA465611 | USP10 is a positive regulator of p53 activity in the DNA damage response, and also acts as a tumor suppressor in cells with wild-type p53. USP10 is also involved in endocytic sorting of ion channels, and has been described as a cofactor for the androgen receptor. | [31,32] | **Detectable** |
| **USP** | USP11 | ubiquitin specific protease 11 | UHX1 | NM_004651 | NP_004642 | IRAUp969E023D | USP11 has been shown to negatively regulate TNFalpha-induced NF-kappaB activity, to be involved in double-strand break DNA repair, and to modulate transcriptional repression through the Polycomb repressive complex 1. | [24,33] | **Detectable** |
| **USP** | USP12 | ubiquitin specific peptidase 12 | USP12L1 | NM_182488 | NP_872294 | IRATp970G0127D | USP12, like USP46, forms a ternary complex with UAF1 and WDR20. The ternary complex is enzymatically active, however, its cellular function is unknown. | [34,35] | **Barely detectable** |
| **USP** | USP13 | ubiquitin specific peptidase 13 (isopeptidase T-3) | ISOT3, IsoT-3 | NM_003940 | NP_003931 | HU3_p983F034D | USP13 hydrolizes the UB-like molecule, ISG15, and has been described as a diagnostic marker in thyroid cancer. | [36,37] | **Undetectable** |
| **USP** | USP14 | ubiquitin specific peptidase 14 (tRNA-guanine transglycosylase) | TGT | NM_005151 | NP_005142 | HU3_p983H12349D | USP14 is a proteasome-associated DUB involved in Ub regeneration and regulation of protein turnover. It is also essential for synaptic development and function at neuromuscular junctions, CXCR4 degradation and chemotaxis, and spermatogenesis. Finally, it is a physiological inhibitor of endoplasmic reticulum-associated degradation (ERAD). | [38-40] | **Detectable** |
| **USP** | USP15 | ubiquitin specific peptidase 15 | UNPH4, KIAA0529, MGC74854, MGC131982, MGC149838 | NM_006313 | NP_006304 | IRAUp969A0385D | USP15 binds to the COP9 signalosome that regulates the Ub-proteasome system. It has been implicated in the regulation of apoptosis, the Wnt/beta-catenin signaling pathway, and stability of the E6 oncoprotein. | [41] | **Detectable** |
| **USP** | USP16 | ubiquitin specific peptidase 16 | UBP-M | NM_006447 | NP_006438 | AA489619 | USP16 is involved in the regulation of cell cycle progression (in particular, mitosis) and gene expression, through its ability to deubiquitinate H2A. | [42,43] | **Detectable** |
| **USP** | USP17 | ubiquitin specific peptidase 17 | RS447, MGC119330, MGC119331, MGC119333 | NM_001105662 | NP_001099132 | IRAMp995C157Q | USP17 is required for cell cycle progression, in particular the G1 to S phase tranistion. It also regulates virus-induced type I IFN signaling and blocks EGF-induced N-Ras membrane trafficking and activation | [44,45] | **Detectable** |
| **USP** | USP18 | ubiquitin specific protease 18 | ISG43, UBP43 | NM_017414 | NP_059110 | IRATp970H0411D | USP18 possesses ISG15 isopeptidase activity, and regulates interferon signalling, as well as EGFR synthesis. | [46,47] | **Detectable** |
| **USP** | USP19 | ubiquitin specific peptidase 19 | ZMYND9 | NM_006677 | NP_006668 | AA455107 | USP19 is involved in the regulation of the cell cycle, transcription of major myofibrillar proteins in muscle, and ERAD substrate turnover. | [48,49] | **Undetectable** |
| **USP** | USP20 | ubiquitin specific protease 20 | VDU2, LSFR3A, KIAA1003 | NM_006676 | NP_006667 | IRAKp961F2234Q | USP20 regulates trafficking of beta2 adrenergic receptor, and deubiquitinates and stabilizes HIF-1alpha. | [50,51] | **Undetectable** |
| **USP** | USP21 | ubiquitin-specific protease 21 | USP16, USP23, MGC3394 | NM_012475 | NP_036607 | H42874 | USP21 is able to cleave both Ub and Nedd8 from target proteins. It is involved in the regulation of gene expression through its action on H2A, and downregulates TNFalpha-induced NF-kappaB activation through the deubiquitination of RIP1. | [52,53] | **Detectable** |
| **USP** | USP22 | ubiquitin specific protease 22 | USP3L, KIAA1063 | NM_015276 | NP_056091 | AI668850 | USP22 is part of the 11-gene “death from cancer signature” that identifies aggressive tumors, possibly rich in cancer stem cells. USP22 is also a subunit of the human SAGA complex that deubiquitinates H2A and H2B. It is required for transcription and cell-cycle progression. | [54] | **Detectable** |
| **USP** | USP24 | ubiquitin specific protease 24 | FLJ31309, KIAA1057 | NM_015306 | NP_056121 | W86608 | USP24 is a candidate gene for late onset Parkinson disease. | [55] | **Detectable** |
| **USP** | USP25 | ubiquitin specific protease 25 | USP21 | NM_013396 | NP_037528 | IRATp970E0512D | USP25 encodes three isoforms; the longest isoform (USP25m) is restricted to muscle tissues, is upregulated during myogenesis, and rescues myosin binding protein C1 from proteasomal degradation. The tyrosine kinase SYK has been shown to phosphorylate and regulate the levels of USP25. | [56,57] | **Detectable** |
| **USP** | USP26 | ubiquitin-specific protease 26 | MGC120066, MGC120067, MGC120068 | NM_031907 | NP_114113 | IRAMp995M145Q | USP26 is highly expressed in human testes and has been implicated in spermatogenesis, testicular development, and male infertility. It also regulated the transcriptional activity of the androgen receptor. | [58,59] | **Undetectable** |
| **USP** | USP28 | ubiquitin specific protease 28 | KIAA1515 | NM_020886 | NP_065937 | IRATp970F0885D | USP28 is involved in the DNA damage checkpoint pathway. Upon DNA damage, it stabilizes Claspin leading to Chk1 activation of G2 arrest, and Chk2 and 53BP1 leading to apoptosis. USP28 also dissociates from MYC, promoting its degradation and cell cycle arrest. | [60,61] | **Detectable** |
| **USP** | USP29 | ubiquitin specific protease 29 | MGC163266, MGC163270, HOM-TES-84/86 | NM_020903 | NP_065954 | HU3_p983A09168D | n.a. |  | **Undetectable** |
| **USP** | USP30 | ubiquitin specific peptidase 30 | FLJ40511, MGC10702 | NM_032663 | NP_116052 | AA872309 | USP30 participates in the maintenance of mitochondrial morphology. | [62] | **Undetectable** |
| **USP** | USP31 | ubiquitin specific peptidase 31 | KIAA1203 | NM_020718 | NP_065769 | W84743 | USP31 may be involved in the regulation of NF-kappaB activation by members of the TNF receptor superfamily. | [63] | **Detectable** |
| **USP** | USP32 | ubiquitin specific protease 32 | USP10, NY-REN-60 | NM_032582 | NP_115971 | IRAKp961P21115Q | USP32 is overexpressed in beast cancers and might be involved in cell proliferation and migration. | [64] | **Barely detectable** |
| **USP** | USP33 | ubiquitin specific protease 33 | VDU1, KIAA1097**,** MGC16868 | NM_015017 | NP_055832 | AA917376 | USP33 regulates cell migration through the Slit-Robo pathway, receptor trafficking, and thyroid hormone activation. | [51,65] | **Undetectable** |
| **USP** | USP34 | ubiquitin specific protease 34 | FLJ43910, KIAA0570, KIAA0729, MGC104459 | NM_014709 | NP_055524 | H93080 | n.a. |  | **Undetectable** |
| **USP** | USP35 | ubiquitin specific protease 35 |  | NM_020798 | NP_065849 | IRAKp961A2371Q | n.a. |  | **Barely detectable** |
| **USP** | USP36 | ubiquitin specific protease 36 | DUB1 | NM_025090 | NP_079366 | AA256385 | USP36 regulates the structure and function of nucleoli. | [66] | **Undetectable** |
| **USP** | USP37 | ubiquitin specific peptidase 37 | KIAA1594, MGC117261 | NM_020935 | NP_065986 | AA705874 | n.a. |  | **Barely detectable** |
| **USP** | USP38 | ubiquitin specific peptidase 38 | FLJ35970, HP43.8KD, KIAA1891 | NM_032557 | NP_115946 | AA101844 | n.a. |  | **Undetectable** |
| **USP** | USP39 | ubiquitin specific protease 39 | SAD1, CGI-21, HSPC332,MGC75069 | NM_006590 | NP_006581 | AA598988 | USP39 is proposed to be involved in splicing of Aurora B and other mRNAs that are essential for proper spindle checkpoint function. | [67] | **Detectable** |
| **USP** | USP40 | ubiquitin specific protease 40 | FLJ10785, FLJ42100 | NM_018218 | NP_060688 | IRAUp969B0449D | USP40 is a candidate gene for late-onset Parkinson disease. | [55] | **Undetectable** |
| **USP** | USP41 | ubiquitin specific protease 41 |  | XM_036729 | XP_036729 | IMAGp998M0510462Q | n.a. |  | **Barely detectable** |
| **USP** | USP42 | ubiquitin specific protease 42 | FLJ12697 | NM_032172 | NP_115548 | AA872041 | n.a. |  | **Barely detectable** |
| **USP** | USP43 | ubiquitin specific protease 43 | FLJ30626 | NM_153210 | NP_694942 | IMAGp998H0613595Q1 | n.a. |  | **Undetectable** |
| **USP** | USP44 | ubiquitin specific protease 44 | FLJ14528, DKFZP434D0127 | NM_032147 | NP_115523 | DKFZp434D0127Q | USP44 is a critical regulator of the spindle checkpoint that control entry into anaphase. | [68] | **Undetectable** |
| **USP** | USP45 | ubiquitin specific peptidase 45 | MGC14793 | NM_001080481 | NP_001073950 | IRAUp969E0645D | n.a. |  | **Undetectable** |
| **USP** | USP46 | ubiquitin specific peptidase 46 | FLJ12552 | NM_022832 | NP_073743 | IRATp970G0355D | USP46, like USP12, forms a ternary complex with UAF1 and WDR20. The ternary complex is enzymatically active, however, the cellular function of this complex is unknown. | [34] | **Undetectable** |
| **USP** | USP47 | ubiquitin specific protease 47 | TRFP, FLJ20727 | NM_017944 | NP_060414 | AA598675 | USP47 interacts with the E3 ligase, beta-TRCP, and is involved in the regulation of cell growth and survival. | [69] | **Undetectable** |
| **USP** | USP48 | ubiquitin specific protease 48 | USP31, RAP1GA1, MGC14879, MGC132556, DKFZp762M1713 | NM_032236 | NP_115612 | IRATp970C0610D | n.a. |  | **Detectable** |
| **USP** | USP49 | ubiquitin specific peptidase 49 | MGC20741 | NM_018561 | NP_061031 | IRAUp969E1068D | n.a. |  | **Detectable** |
| **USP** | USP50 | ubiquitin specific protease 50 |  | NM_203494 | NP_987090 | IMAGp998B116219Q | n.a. |  | **Undetectable** |
| **USP** | USP51 | ubiquitin specific protease 51 |  | NM_201286 | NP_958443 | HU3_p983E05342D | n.a. |  | **Undetectable** |
| **USP** | USP53 | ubiquitin specific protease 53 | DKFZp781E1417 | NM_019050 | NP_061923 | AI276062 | n.a. |  | **Undetectable** |
| **USP** | USP54 | ubiquitin specific peptidase 54 | C10orf29, FLJ37318, bA137L10.3, bA137L10.4 | NM_152586 | NP_689799 | IRAKp961K04140Q | n.a. |  | **Undetectable** |
| **USP** | USPL1 | ubiquitin specific peptidase like 1 | C13orf22, D13S106E, FLJ32952, bA121O19.1, DKFZp781K2286, RP11-121O19.1 | NM_005800 | NP_005791 | IRATp970G0629D | n.a. |  | **Undetectable** |
| **OTUBAIN** | OTUB1 | OTU domain, ubiquitin aldehyde binding 1 | OTB1, OTU1, FLJ20113, FLJ40710, MGC111158 | NM_017670 | NP_060140 | AI289139 | OTUB1 negatively regulates DNA damage repair, virus-triggered type I IFN induction and cellular antiviral response, and estrogen receptor alpha transcriptional activity. | [70,71] | **Detectable** |
| **OTUBAIN** | OTUB2 | OTU domain, ubiquitin aldehyde binding 2 | OTB2, OTU2, MGC3102, FLJ21916, C14orf137 | NM_023112 | NP_075601 | H10030 | OTUB2 negatively regulates virus-triggered type I IFN induction and cellular antiviral response by deubiquitinating TRAF3 and -6. | [70] | **Barely detectable** |
| **OTUBAIN** | OTUD1 | OTU domain containing 1 | DUBA7, OTDC1 | NM_001145373 | NP_001138845 | HU3_p983B04254D | n.a. |  | **Undetectable** |
| **OTUBAIN** | OTUD3 | OTU domain containing 3 | DUBA4, KIAA0459, RP11-460G22.1 | NM_015207 | NP_056022 | H15653 | OTUD3 might act as a negative regulator of innate immune responses. | [72] | **Undetectable** |
| **OTUBAIN** | OTUD4 | OTU domain containing 4 | HIN1, HSHIN1, KIAA1046, DKFZp434I0721 | NM_199324 | NP_955356 | R78541 | n.a. |  | **Undetectable** |
| **OTUBAIN** | OTUD5 | OTU domain containing 5 | DUBA, MGC104871, DKFZp761A052 | NM_017602 | NP_060072 | IRATp970E0950D | OTUD5 regulates type I interferon production. | [72] | **Barely detectable** |
| **OTUBAIN** | OTUD6B | OTU domain containing 6B | DUBA5, CGI-77 | NM_016023 | NP_057107 | H44448 | n.a. |  | **Undetectable** |
| **OTUBAIN** | OTUD7A | OTU domain containing 7A | C15orf16, CEZANNE2 | NM_130901 | NP_570971 | IRAKp961I1179Q | n.a. |  | **Barely detectable** |
| **OTUBAIN** | OTUD7B | OTU domain containing 7B | ZA20D1, CEZANNE | NM_020205 | NP_064590 | IRAUp969H0984D | OTUD7B inhibits NF-kappa B activation. | [73] | **Undetectable** |
| **OTUBAIN** | TNFAIP3 | tumor necrosis factor, alpha-induced protein 3 | A20, OTUD7C, TNFA1P2, MGC104522, MGC138687, MGC138688 | NM_006290 | NP_006281 | AA476272 | TNFAIP3 is a key negative regulator of inflammation and immunity. It is thought to act as tumor suppressor in B-cell lymphomas, and has been implicated in autoimmune diseases. | [74] | **Undetectable** |
| **OTUBAIN** | VCPIP1 | valosin containing protein (p97)/p47 complex interacting protein 1 | DUB3A, VCIP135, FLJ23132, KIAA1850, DKFZp686G038 | NM_025054 | NP_079330 | AA405241 | VCPIP1 regulates reassembly of Golgi stacks after mitosis. | [75] | **Barely detectable** |
| **OTUBAIN** | YOD1 | YOD1 OTU deubiquinating enzyme 1 homolog | DUBA8, OTUD2, PRO0907, DKFZp451J1719, RP11-164O23.1, YOD1 | NM_018566 | NP_061036 | HU3_p983G03353D | YOD1 forms a complex with p97, NPL4 and UFD1, which is involved in the dislocation of misfolded proteins from the ER. | [76] | **Undetectable** |
| **OTUBAIN** | ZRANB1 | zinc finger, RAN-binding domain containing 1 protein | TRABID, DKFZp762P2216 | NM_017580 | NP_060050 | IRAKp961H1593Q | ZRANB1 is a positive regulator of the Wnt signaling pathway that acts by deubiquitinating APC protein. | [77] | **Barely detectable** |
| **MACHADO** | ATXN3 | ataxin 3 | AT3, ATX3, JOS, MJD, MJD1, SCA3 | NM_004993 | NP_004984 | IRATp970F0352D | Expansion of a polyglutamine tract in ATXN3 causes Machado-Joseph disease, a late-onset neurodegenerative disorder. ATXN3 appears to be involved in the organisation of the cytoskeleton | [78,79] | **Undetectable** |
| **MACHADO** | ATXN3L | ataxin 3-like | MJDL, FLJ59638, MGC168806, MGC168807 | NM_001135995 | NP_001129467 | IMAGp998O091784Q | n.a. |  | **Undetectable** |
| **MACHADO** | JOSD1 | Josephin domain containing 1 | KIAA0063, dJ508I15.2 | NM_014876 | NP_055691 | T63072 | n.a. |  | **Detectable** |
| **MACHADO** | JOSD2 | Josephin domain containing 2 | SBBI54, FLJ29018 | NM_138334 | NP_612207 | AA670415 | n.a. |  | **Undetectable** |
| **MACHADO** | JOSD3 | TATA box binding protein (TBP)-associated factor, RNA polymerase I, D, 41kDa | MGC5306, TAF1D | NM_024116 | NP_077021 | AA453287 | JOSD3 has a role in RNA polymerase I transcription. | [80] | **Undetectable** |
| **JAMM** | BRCC3 | BRCA1/BRCA2-containing complex, subunit 3 | C6.1A, BRCC36, CXorf53, RP11-143H17.2 | NM_024332 | NP_077308 | IRAUp969D1018D | BRCC3 is involved in DNA repair pathways. | [81,82] | **Undetectable** |
| **JAMM** | COPS5 | COP9 constitutive photomorphogenic homolog subunit 5 (Arabidopsis) | CSN5, JAB1, SGN5, MOV-34, MGC3149 | NM_006837 | NP_006828 | AA460599 | COPS5 is a subunit of the COP9 signalosome that regulates the Ub-proteasome system. It has been implicated in the regulation of many proteins including p53, c-Jun, cyclin E, p27kip1, SCF ubiquitin ligase and Smad family proteins. It is also involved in exosomal protein sorting. | [83] | **Detectable** |
| **JAMM** | COPS6 | COP9 constitutive photomorphogenic homolog subunit 6 (Arabidopsis) | CSN6, MOV34-34KD | NM_006833 | NP_006824 | AA992441 | COPS6 is a subunit of the COP9 signalosome that regulates the Ub-proteasome system. COPS6 protein belongs to the translation initiation factor 3 (eIF3) superfamily. It is involved in the regulation of cell cycle and is likely to be a cellular cofactor for HIV-1 accessory gene product Vpr | [84] | **Detectable** |
| **JAMM** | EIF3F | eukaryotic translation initiation factor 3, subunit F | EIF3S5, eIF3-p47 | NM_003754 | NP_003745 | AI650470 | EIF3F is a component of the eukaryotic translation initiation factor 3 (eIF-3) complex, which is required for protein synthesis. It also specifically interferes with the 3' end processing of HIV-1 mRNAs | [85] | **Detectable** |
| **JAMM** | EIF3H | eukaryotic translation initiation factor 3, subunit H | EIF3S3, eIF3-p40, MGC102958, eIF3-gamma | NM_003756 | NP_003747 | AI017703 | EIF3H is a component of the eukaryotic translation initiation factor 3 (eIF-3) complex, which is required for protein synthesis. High eIF3h levels directly stimulate protein synthesis, resulting in the establishment and maintenance of the malignant state in cells. | [86] | **Detectable** |
| **JAMM** | MPND | MPN domain containing | FLJ14981 | NM_032868 | NP_116257 | IRATp970G0855D | n.a. |  | **Undetectable** |
| **JAMM** | MYSM1 | Myb-like, SWIRM and MPN domains 1 | 2A-DUB, KIAA1915, RP4-592A1.1, DKFZp779J1554,DKFZp779J1721 | NM_001085487 | NP_001078956 | HU3_p983F08226D | MYSM1 regulates transcription by deubiquitinating monoubiquitinated H2A. | [87] | **Barely detectable** |
| **JAMM** | PRPF8 | PRP8 pre-mRNA processing factor 8 homolog (S. cerevisiae) | PRP8, RP13, HPRP8, PRPC8 | NM_006445 | NP_006436 | IRATp970C0384D | PRPF8 is a central component of the spliceosome. It is also a candidate gene for autosomal dominant retinitis pigmentosa. | [88,89] | **Detectable** |
| **JAMM** | PSMD14 | proteasome (prosome, macropain) 26S subunit, non-ATPase, 14 | PAD1, POH1, rpn11 | NM_005805 | NP_005796 | IRATp970F129D | PSMD14 is a component of the 26S proteasome that has been shown to regulate c-Jun and ErbB2 levels. | [90,91] | **Detectable** |
| **JAMM** | PSMD7 | proteasome (prosome, macropain) 26S subunit, non-ATPase, 7 | P40, S12, Rpn8, MOV34 | NM_002811 | NP_002802 | IRAUp969D1054D | PMSD7 is a 26S proteasome subunit with no isopeptidase activity | [92] | **Detectable** |
| **JAMM** | STAMBP | STAM binding protein | AMSH, MGC126516, MGC126518 | NM_006463 | NP_006454 | AA454618 | STAMBP is an ESCRT-III associated enzyme that deubiquitinates cargo on MVB/late endosomes preceding lysosomal degradation | [93,94] | **Detectable** |
| **JAMM** | STAMBPL1 | STAM binding protein-like 1 | AMSH-FP, AMSH-LP, ALMalpha, FLJ31524, KIAA1373, bA399O19.2 | NM_020799 | NP_065850 | HU3_p983B107D | STAMBPL1 is an AMSH-like protein that does not bind the SH3 domain of STAM1 protein | [95] | **Barely detectable** |

1. Nishikawa H, Wu W, Koike A, Kojima R, Gomi H, et al. (2009) BRCA1-associated protein 1 interferes with BRCA1/BARD1 RING heterodimer activity. Cancer Res 69: 111-119.

2. Yu H, Mashtalir N, Daou S, Hammond-Martel I, Ross J, et al. (2010) The Ubiquitin Carboxyl Hydrolase BAP1 Forms a Ternary Complex with YY1 and HCF-1 and is a Critical Regulator of Gene Expression. Mol Cell Biol.

3. Gong B, Leznik E (2007) The role of ubiquitin C-terminal hydrolase L1 in neurodegenerative disorders. Drug News Perspect 20: 365-370.

4. Fang Y, Fu D, Shen XZ (2010) The potential role of ubiquitin c-terminal hydrolases in oncogenesis. Biochim Biophys Acta 1806: 1-6.

5. Butterworth MB, Edinger RS, Ovaa H, Burg D, Johnson JP, et al. (2007) The deubiquitinating enzyme UCH-L3 regulates the apical membrane recycling of the epithelial sodium channel. J Biol Chem 282: 37885-37893.

6. Yao T, Song L, Jin J, Cai Y, Takahashi H, et al. (2008) Distinct modes of regulation of the Uch37 deubiquitinating enzyme in the proteasome and in the Ino80 chromatin-remodeling complex. Mol Cell 31: 909-917.

7. Wicks SJ, Haros K, Maillard M, Song L, Cohen RE, et al. (2005) The deubiquitinating enzyme UCH37 interacts with Smads and regulates TGF-beta signalling. Oncogene 24: 8080-8084.

8. Courtois G (2008) Tumor suppressor CYLD: negative regulation of NF-kappaB signaling and more. Cell Mol Life Sci 65: 1123-1132.

9. Tauriello DV, Haegebarth A, Kuper I, Edelmann MJ, Henraat M, et al. Loss of the tumor suppressor CYLD enhances Wnt/beta-catenin signaling through K63-linked ubiquitination of Dvl. Mol Cell 37: 607-619.

10. Uchida N, Hoshino S, Katada T (2004) Identification of a human cytoplasmic poly(A) nuclease complex stimulated by poly(A)-binding protein. J Biol Chem 279: 1383-1391.

11. Nijman SM, Huang TT, Dirac AM, Brummelkamp TR, Kerkhoven RM, et al. (2005) The deubiquitinating enzyme USP1 regulates the Fanconi anemia pathway. Mol Cell 17: 331-339.

12. Parmar K, Kim J, Sykes SM, Shimamura A, Stuckert P, et al. Hematopoietic stem cell defects in mice with deficiency of Fancd2 or Usp1. Stem Cells 28: 1186-1195.

13. Stevenson LF, Sparks A, Allende-Vega N, Xirodimas DP, Lane DP, et al. (2007) The deubiquitinating enzyme USP2a regulates the p53 pathway by targeting Mdm2. Embo J 26: 976-986.

14. Shan J, Zhao W, Gu W (2009) Suppression of cancer cell growth by promoting cyclin D1 degradation. Mol Cell 36: 469-476.

15. Nicassio F, Corrado N, Vissers JH, Areces LB, Bergink S, et al. (2007) Human USP3 Is a Chromatin Modifier Required for S Phase Progression and Genome Stability. Curr Biol 17: 1972-1977.

16. Wada K, Kamitani T (2006) UnpEL/Usp4 is ubiquitinated by Ro52 and deubiquitinated by itself. Biochem Biophys Res Commun 342: 253-258.

17. Wada K, Tanji K, Kamitani T (2006) Oncogenic protein UnpEL/Usp4 deubiquitinates Ro52 by its isopeptidase activity. Biochem Biophys Res Commun 339: 731-736.

18. Dayal S, Sparks A, Jacob J, Allende-Vega N, Lane DP, et al. (2009) Suppression of the deubiquitinating enzyme USP5 causes the accumulation of unanchored polyubiquitin and the activation of p53. J Biol Chem 284: 5030-5041.

19. Masuda-Robens JM, Kutney SN, Qi H, Chou MM (2003) The TRE17 oncogene encodes a component of a novel effector pathway for Rho GTPases Cdc42 and Rac1 and stimulates actin remodeling. Mol Cell Biol 23: 2151-2161.

20. Bizimungu C, Thomas A, Brasseur R, Vandenbol M (2007) Mutational analysis of the TRE2 oncogene encoding an inactive RabGAP. Biotechnol Lett 29: 1927-1937.

21. Martinu L, Masuda-Robens JM, Robertson SE, Santy LC, Casanova JE, et al. (2004) The TBC (Tre-2/Bub2/Cdc16) domain protein TRE17 regulates plasma membrane-endosomal trafficking through activation of Arf6. Mol Cell Biol 24: 9752-9762.

22. Li M, Chen D, Shiloh A, Luo J, Nikolaev AY, et al. (2002) Deubiquitination of p53 by HAUSP is an important pathway for p53 stabilization. Nature 416: 648-653.

23. Brooks CL, Gu W (2004) Dynamics in the p53-Mdm2 ubiquitination pathway. Cell Cycle 3: 895-899.

24. Maertens GN, El Messaoudi-Aubert S, Elderkin S, Hiom K, Peters G (2010) Ubiquitin-specific proteases 7 and 11 modulate Polycomb regulation of the INK4a tumour suppressor. Embo J 29: 2553-2565.

25. Mizuno E, Kobayashi K, Yamamoto A, Kitamura N, Komada M (2006) A deubiquitinating enzyme UBPY regulates the level of protein ubiquitination on endosomes. Traffic 7: 1017-1031.

26. Niendorf S, Oksche A, Kisser A, Lohler J, Prinz M, et al. (2007) Essential role of ubiquitin-specific protease 8 for receptor tyrosine kinase stability and endocytic trafficking in vivo. Mol Cell Biol 27: 5029-5039.

27. Dupont S, Mamidi A, Cordenonsi M, Montagner M, Zacchigna L, et al. (2009) FAM/USP9x, a deubiquitinating enzyme essential for TGFbeta signaling, controls Smad4 monoubiquitination. Cell 136: 123-135.

28. Schwickart M, Huang X, Lill JR, Liu J, Ferrando R, et al. (2010) Deubiquitinase USP9X stabilizes MCL1 and promotes tumour cell survival. Nature 463: 103-107.

29. Hopps CV, Mielnik A, Goldstein M, Palermo GD, Rosenwaks Z, et al. (2003) Detection of sperm in men with Y chromosome microdeletions of the AZFa, AZFb and AZFc regions. Hum Reprod 18: 1660-1665.

30. Tyler-Smith C, Krausz C (2009) The will-o'-the-wisp of genetics--hunting for the azoospermia factor gene. N Engl J Med 360: 925-927.

31. Yuan J, Luo K, Zhang L, Cheville JC, Lou Z (2010) USP10 Regulates p53 Localization and Stability by Deubiquitinating p53. Cell 140: 384-396.

32. Bomberger JM, Barnaby RL, Stanton BA (2009) The deubiquitinating enzyme USP10 regulates the post-endocytic sorting of cystic fibrosis transmembrane conductance regulator in airway epithelial cells. J Biol Chem 284: 18778-18789.

33. Yamaguchi T, Kimura J, Miki Y, Yoshida K (2007) The deubiquitinating enzyme USP11 controls an IkappaB kinase alpha (IKKalpha)-p53 signaling pathway in response to tumor necrosis factor alpha (TNFalpha). J Biol Chem 282: 33943-33948.

34. Cohn MA, Kee Y, Haas W, Gygi SP, D'Andrea AD (2009) UAF1 is a subunit of multiple deubiquitinating enzyme complexes. J Biol Chem 284: 5343-5351.

35. Kee Y, Yang K, Cohn MA, Haas W, Gygi SP, et al. WDR20 regulates activity of the USP12 x UAF1 deubiquitinating enzyme complex. J Biol Chem 285: 11252-11257.

36. Catic A, Fiebiger E, Korbel GA, Blom D, Galardy PJ, et al. (2007) Screen for ISG15-crossreactive deubiquitinases. PLoS One 2: e679.

37. Fontaine JF, Mirebeau-Prunier D, Raharijaona M, Franc B, Triau S, et al. (2009) Increasing the number of thyroid lesions classes in microarray analysis improves the relevance of diagnostic markers. PLoS One 4: e7632.

38. Crimmins S, Jin Y, Wheeler C, Huffman AK, Chapman C, et al. (2006) Transgenic rescue of ataxia mice with neuronal-specific expression of ubiquitin-specific protease 14. J Neurosci 26: 11423-11431.

39. Mines MA, Goodwin JS, Limbird LE, Cui FF, Fan GH (2009) Deubiquitination of CXCR4 by USP14 is critical for both CXCL12-induced CXCR4 degradation and chemotaxis but not ERK ativation. J Biol Chem 284: 5742-5752.

40. Lee BH, Lee MJ, Park S, Oh DC, Elsasser S, et al. Enhancement of proteasome activity by a small-molecule inhibitor of USP14. Nature 467: 179-184.

41. Xu M, Takanashi M, Oikawa K, Tanaka M, Nishi H, et al. (2009) USP15 plays an essential role for caspase-3 activation during Paclitaxel-induced apoptosis. Biochem Biophys Res Commun 388: 366-371.

42. Joo HY, Zhai L, Yang C, Nie S, Erdjument-Bromage H, et al. (2007) Regulation of cell cycle progression and gene expression by H2A deubiquitination. Nature 449: 1068-1072.

43. Shanbhag NM, Rafalska-Metcalf IU, Balane-Bolivar C, Janicki SM, Greenberg RA (2010) ATM-dependent chromatin changes silence transcription in cis to DNA double-strand breaks. Cell 141: 970-981.

44. de la Vega M, Burrows JF, McFarlane C, Govender U, Scott CJ, et al. (2010) The deubiquitinating enzyme USP17 blocks N-Ras membrane trafficking and activation but leaves K-Ras unaffected. J Biol Chem 285: 12028-12036.

45. McFarlane C, Kelvin AA, de la Vega M, Govender U, Scott CJ, et al. (2010) The deubiquitinating enzyme USP17 is highly expressed in tumor biopsies, is cell cycle regulated, and is required for G1-S progression. Cancer Res 70: 3329-3339.

46. Malakhova OA, Kim KI, Luo JK, Zou W, Kumar KG, et al. (2006) UBP43 is a novel regulator of interferon signaling independent of its ISG15 isopeptidase activity. Embo J 25: 2358-2367.

47. Potu H, Sgorbissa A, Brancolini C (2010) Identification of USP18 as an important regulator of the susceptibility to IFN-alpha and drug-induced apoptosis. Cancer Res 70: 655-665.

48. Lu Y, Adegoke OA, Nepveu A, Nakayama KI, Bedard N, et al. (2009) USP19 deubiquitinating enzyme supports cell proliferation by stabilizing KPC1, a ubiquitin ligase for p27Kip1. Mol Cell Biol 29: 547-558.

49. Hassink GC, Zhao B, Sompallae R, Altun M, Gastaldello S, et al. (2009) The ER-resident ubiquitin-specific protease 19 participates in the UPR and rescues ERAD substrates. EMBO Rep 10: 755-761.

50. Li Z, Wang D, Messing EM, Wu G (2005) VHL protein-interacting deubiquitinating enzyme 2 deubiquitinates and stabilizes HIF-1alpha. EMBO Rep 6: 373-378.

51. Berthouze M, Venkataramanan V, Li Y, Shenoy SK (2009) The deubiquitinases USP33 and USP20 coordinate beta2 adrenergic receptor recycling and resensitization. Embo J 28: 1684-1696.

52. Xu G, Tan X, Wang H, Sun W, Shi Y, et al. (2010) Ubiquitin-specific peptidase 21 inhibits tumor necrosis factor alpha-induced nuclear factor kappaB activation via binding to and deubiquitinating receptor-interacting protein 1. J Biol Chem 285: 969-978.

53. Nakagawa T, Kajitani T, Togo S, Masuko N, Ohdan H, et al. (2008) Deubiquitylation of histone H2A activates transcriptional initiation via trans-histone cross-talk with H3K4 di- and trimethylation. Genes Dev 22: 37-49.

54. Zhang XY, Varthi M, Sykes SM, Phillips C, Warzecha C, et al. (2008) The putative cancer stem cell marker USP22 is a subunit of the human SAGA complex required for activated transcription and cell-cycle progression. Mol Cell 29: 102-111.

55. Li Y, Schrodi S, Rowland C, Tacey K, Catanese J, et al. (2006) Genetic evidence for ubiquitin-specific proteases USP24 and USP40 as candidate genes for late-onset Parkinson disease. Hum Mutat 27: 1017-1023.

56. Denuc A, Bosch-Comas A, Gonzalez-Duarte R, Marfany G (2009) The UBA-UIM domains of the USP25 regulate the enzyme ubiquitination state and modulate substrate recognition. PLoS One 4: e5571.

57. Cholay M, Reverdy C, Benarous R, Colland F, Daviet L (2010) Functional interaction between the ubiquitin-specific protease 25 and the SYK tyrosine kinase. Exp Cell Res 316: 667-675.

58. Zhang J, Qiu SD, Li SB, Zhou DX, Tian H, et al. (2007) Novel mutations in ubiquitin-specific protease 26 gene might cause spermatogenesis impairment and male infertility. Asian J Androl 9: 809-814.

59. Dirac AM, Bernards R The deubiquitinating enzyme USP26 is a regulator of androgen receptor signaling. Mol Cancer Res 8: 844-854.

60. Zhang D, Zaugg K, Mak TW, Elledge SJ (2006) A role for the deubiquitinating enzyme USP28 in control of the DNA-damage response. Cell 126: 529-542.

61. Popov N, Wanzel M, Madiredjo M, Zhang D, Beijersbergen R, et al. (2007) The ubiquitin-specific protease USP28 is required for MYC stability. Nat Cell Biol 9: 765-774.

62. Nakamura N, Hirose S (2008) Regulation of mitochondrial morphology by USP30, a deubiquitinating enzyme present in the mitochondrial outer membrane. Mol Biol Cell 19: 1903-1911.

63. Tzimas C, Michailidou G, Arsenakis M, Kieff E, Mosialos G, et al. (2006) Human ubiquitin specific protease 31 is a deubiquitinating enzyme implicated in activation of nuclear factor-kappaB. Cell Signal 18: 83-92.

64. Akhavantabasi S, Akman HB, Sapmaz A, Keller J, Petty EM, et al. (2010) USP32 is an active, membrane-bound ubiquitin protease overexpressed in breast cancers. Mamm Genome 21: 388-397.

65. Yuasa-Kawada J, Kinoshita-Kawada M, Wu G, Rao Y, Wu JY (2009) Midline crossing and Slit responsiveness of commissural axons require USP33. Nat Neurosci 12: 1087-1089.

66. Endo A, Kitamura N, Komada M (2009) Nucleophosmin/B23 regulates ubiquitin dynamics in nucleoli by recruiting deubiquitylating enzyme USP36. J Biol Chem 284: 27918-27923.

67. van Leuken RJ, Luna-Vargas MP, Sixma TK, Wolthuis RM, Medema RH (2008) Usp39 is essential for mitotic spindle checkpoint integrity and controls mRNA-levels of aurora B. Cell Cycle 7: 2710-2719.

68. Stegmeier F, Rape M, Draviam VM, Nalepa G, Sowa ME, et al. (2007) Anaphase initiation is regulated by antagonistic ubiquitination and deubiquitination activities. Nature 446: 876-881.

69. Peschiaroli A, Skaar JR, Pagano M, Melino G (2010) The ubiquitin-specific protease USP47 is a novel beta-TRCP interactor regulating cell survival. Oncogene 29: 1384-1393.

70. Li S, Zheng H, Mao AP, Zhong B, Li Y, et al. (2010) Regulation of virus-triggered signaling by OTUB1- and OTUB2-mediated deubiquitination of TRAF3 and TRAF6. J Biol Chem 285: 4291-4297.

71. Nakada S, Tai I, Panier S, Al-Hakim A, Iemura S, et al. (2010) Non-canonical inhibition of DNA damage-dependent ubiquitination by OTUB1. Nature 466: 941-946.

72. Kayagaki N, Phung Q, Chan S, Chaudhari R, Quan C, et al. (2007) DUBA: a deubiquitinase that regulates type I interferon production. Science 318: 1628-1632.

73. Enesa K, Zakkar M, Chaudhury H, Luong le A, Rawlinson L, et al. (2008) NF-kappaB suppression by the deubiquitinating enzyme Cezanne: a novel negative feedback loop in pro-inflammatory signaling. J Biol Chem 283: 7036-7045.

74. Wertz IE, O'Rourke KM, Zhou H, Eby M, Aravind L, et al. (2004) De-ubiquitination and ubiquitin ligase domains of A20 downregulate NF-kappaB signalling. Nature 430: 694-699.

75. Wang Y, Satoh A, Warren G, Meyer HH (2004) VCIP135 acts as a deubiquitinating enzyme during p97-p47-mediated reassembly of mitotic Golgi fragments. J Cell Biol 164: 973-978.

76. Ernst R, Mueller B, Ploegh HL, Schlieker C (2009) The otubain YOD1 is a deubiquitinating enzyme that associates with p97 to facilitate protein dislocation from the ER. Mol Cell 36: 28-38.

77. Tran H, Hamada F, Schwarz-Romond T, Bienz M (2008) Trabid, a new positive regulator of Wnt-induced transcription with preference for binding and cleaving K63-linked ubiquitin chains. Genes Dev 22: 528-542.

78. Burnett B, Li F, Pittman RN (2003) The polyglutamine neurodegenerative protein ataxin-3 binds polyubiquitylated proteins and has ubiquitin protease activity. Hum Mol Genet 12: 3195-3205.

79. Rodrigues AJ, do Carmo Costa M, Silva TL, Ferreira D, Bajanca F, et al. (2010) Absence of ataxin-3 leads to cytoskeletal disorganization and increased cell death. Biochim Biophys Acta 1803: 1154-1163.

80. Gorski JJ, Pathak S, Panov K, Kasciukovic T, Panova T, et al. (2007) A novel TBP-associated factor of SL1 functions in RNA polymerase I transcription. Embo J 26: 1560-1568.

81. Wang B, Elledge SJ (2007) Ubc13/Rnf8 ubiquitin ligases control foci formation of the Rap80/Abraxas/Brca1/Brcc36 complex in response to DNA damage. Proc Natl Acad Sci U S A 104: 20759-20763.

82. Shao G, Lilli DR, Patterson-Fortin J, Coleman KA, Morrissey DE, et al. (2009) The Rap80-BRCC36 de-ubiquitinating enzyme complex antagonizes RNF8-Ubc13-dependent ubiquitination events at DNA double strand breaks. Proc Natl Acad Sci U S A 106: 3166-3171.

83. Zhang XC, Chen J, Su CH, Yang HY, Lee MH (2008) Roles for CSN5 in control of p53/MDM2 activities. J Cell Biochem 103: 1219-1230.

84. Hrecka K, Gierszewska M, Srivastava S, Kozaczkiewicz L, Swanson SK, et al. (2007) Lentiviral Vpr usurps Cul4-DDB1[VprBP] E3 ubiquitin ligase to modulate cell cycle. Proc Natl Acad Sci U S A 104: 11778-11783.

85. Cappuzzo F, Varella-Garcia M, Rossi E, Gajapathy S, Valente M, et al. (2009) MYC and EIF3H Coamplification significantly improve response and survival of non-small cell lung cancer patients (NSCLC) treated with gefitinib. J Thorac Oncol 4: 472-478.

86. Zhang L, Smit-McBride Z, Pan X, Rheinhardt J, Hershey JW (2008) An oncogenic role for the phosphorylated h-subunit of human translation initiation factor eIF3. J Biol Chem 283: 24047-24060.

87. Zhu P, Zhou W, Wang J, Puc J, Ohgi KA, et al. (2007) A histone H2A deubiquitinase complex coordinating histone acetylation and H1 dissociation in transcriptional regulation. Mol Cell 27: 609-621.

88. Pena V, Liu S, Bujnicki JM, Luhrmann R, Wahl MC (2007) Structure of a multipartite protein-protein interaction domain in splicing factor prp8 and its link to retinitis pigmentosa. Mol Cell 25: 615-624.

89. Towns KV, Kipioti A, Long V, McKibbin M, Maubaret C, et al. (2010) Prognosis for splicing factor PRPF8 retinitis pigmentosa, novel mutations and correlation between human and yeast phenotypes. Hum Mutat 31: E1361-1376.

90. Nabhan JF, Ribeiro P (2006) The 19 S proteasomal subunit POH1 contributes to the regulation of c-Jun ubiquitination, stability, and subcellular localization. J Biol Chem 281: 16099-16107.

91. Liu H, Buus R, Clague MJ, Urbe S (2009) Regulation of ErbB2 receptor status by the proteasomal DUB POH1. PLoS One 4: e5544.

92. Sanches M, Alves BS, Zanchin NI, Guimaraes BG (2007) The crystal structure of the human Mov34 MPN domain reveals a metal-free dimer. J Mol Biol 370: 846-855.

93. Agromayor M, Martin-Serrano J (2006) Interaction of AMSH with ESCRT-III and deubiquitination of endosomal cargo. J Biol Chem 281: 23083-23091.

94. Kyuuma M, Kikuchi K, Kojima K, Sugawara Y, Sato M, et al. (2007) AMSH, an ESCRT-III associated enzyme, deubiquitinates cargo on MVB/late endosomes. Cell Struct Funct 31: 159-172.

95. Kikuchi K, Ishii N, Asao H, Sugamura K (2003) Identification of AMSH-LP containing a Jab1/MPN domain metalloenzyme motif. Biochem Biophys Res Commun 306: 637-643.
