# Supplementary material for: An Atlas of Altered Expression of Deubiquitinating Enzymes in Human Cancer
Source: PLoS One. 2011 Jan 25;6(1):e15891. doi: 10.1371/journal.pone.0015891 (PMC3026797; doi:10.1371/journal.pone.0015891)
Supplement: Table S4 — The clinical and pathological information for the patients of the NSCLC cohort is reported. For some patients not all information was available (No data). *For three patients no follow-up was available. (DOC) [file pone.0015891.s005.doc]

**Table S4. Clinical and pathological information of the NSCLC cohort**

| **Parameter** | **Group** | **NSCLC COHORT (N = 420)** | |
| --- | --- | --- | --- |
|  |  | **N** | **%** |
| **Gender** | *Female* | 97 | 23.1 |
|  | *Male* | 323 | 76.9 |
| **Age** | *<65* | 179 | 42.6 |
|  | *≥65* | 241 | 57.4 |
| **Histotype** | *Adenocarcinoma (ACC)* | 244 | 58.1 |
|  | *Squamous cell carcinoma (SCC)* | 176 | 41.9 |
| **Ki67** | *<20% (Neg.)* | 165 | 45.5 |
|  | *≥20% (Pos.)* | 198 | 54.5 |
|  | *No data* | 57 |  |
| **p53** | *≤15% (Neg.)* | 176 | 48.9 |
|  | *>15% (Pos.)* | 184 | 51.1 |
|  | *No data* | 60 |  |
| **Grade** | *G1* | 41 | 9.9 |
|  | *G2* | 163 | 39.5 |
|  | *G3* | 209 | 50.6 |
|  | *No data* | 7 |  |
| **pT** | *1-2* | 335 | 79.8 |
|  | *3-4* | 85 | 20.2 |
| **Nodal Status** | *Neg* | 229 | 54.5 |
|  | *Pos* | 191 | 45.5 |
| **Stage** | *1a 1b* | 191 | 54.5 |
|  | *2+* | 229 | 54.5 |
| **Stage** | *1a 1b* | 191 | 45.5 |
|  | *2a 2b* | 158 | 37.6 |
|  | *3a 3b 4* | 71 | 16.9 |
| **Death any causes** | *No* | 179 | 42.9 |
|  | *Yes* | 238 | 57.1 |
|  | *No data* | 3 |  |
| **Death any causes (within 5 years)*** | *No* | 217 | 52.0 |
|  | *Yes* | 200 | 48.0 |
| **Death any causes (within 7 years)*** | *No* | 187 | 44.8 |
|  | *Yes* | 230 | 55.2 |
