# Supplementary material for: An Atlas of Altered Expression of Deubiquitinating Enzymes in Human Cancer
Source: PLoS One. 2011 Jan 25;6(1):e15891. doi: 10.1371/journal.pone.0015891 (PMC3026797; doi:10.1371/journal.pone.0015891)
Supplement: Table S5 — The clinical and pathological information for the patients of the melanoma cohort is shown. Clinical parameters are reported only for the 138 primary melanomas. For some patients not all information was available (No data). Histotypes: NM, Nodular Melanoma; SSM, Superficial Spreading Melanoma; TIL: Tumor-Infiltrating Lymphocytes. (DOC) [file pone.0015891.s006.doc]

**Table S5. Clinical and pathological information of the melanoma cohort**

| **Parameter** | **Group** | **Melanoma Cohort (N=232)** | |
| --- | --- | --- | --- |
|  |  | **N** | **%** |
| **Type** | *Nevi* | 32 | 13.8 |
|  | *Melanoma* | 138 | 59.5 |
|  | *Metastatic Melanoma* | 62 | 26.7 |
| **Gender** | *FEMALE* | 56 | 40.9 |
|  | *MALE* | 81 | 59.1 |
|  | *No data* | 1 |  |
| **Age** | *<65* | 95 | 71.4 |
|  | *≥55* | 38 | 28.6 |
|  | *No data* | 5 |  |
| **Mitotic Count** | *0-1* | 51 | 42.5 |
|  | *2-6* | 44 | 36.7 |
|  | *>6* | 25 | 20.8 |
|  | *No data* | 18 |  |
| **Breslow** | *0-1* | 70 | 51.5 |
|  | *2-3* | 39 | 28.7 |
|  | *3+* | 27 | 19.8 |
|  | *No data* | 2 |  |
| **Clark** | *1-2* | 42 | 31.1 |
|  | *3-5* | 93 | 68.9 |
|  | *No data* | 3 |  |
| **Histotype** | *NM* | 16 | 11.9 |
|  | *SSM* | 118 | 88.1 |
|  | *No data* | 4 |  |
| **pT** | *1 (1, 1b, 1c)* | 30 | 32.3 |
|  | *2-4 (2, 2a, 2b, 3, 3a, 3b, 4, 4a)* | 63 | 67.7 |
|  | *No data* | 45 |  |
| **Nodal Status** | *Neg* | 41 | 77.4 |
|  | *Pos* | 12 | 22.6 |
|  | *No data* | 85 |  |
| **Regression** | *No* | 97 | 72.4 |
|  | *Yes* | 37 | 27.6 |
|  | *No data* | 4 |  |
| **Ulceration** | *No* | 99 | 73.3 |
|  | *Yes* | 36 | 26.7 |
|  | *No data* | 3 |  |
| **TIL** | *No* | 86 | 63.7 |
|  | *Yes* | 49 | 36.3 |
|  | *No data* | 3 |  |
