# Supplementary material for: An Atlas of Altered Expression of Deubiquitinating Enzymes in Human Cancer
Source: PLoS One. 2011 Jan 25;6(1):e15891. doi: 10.1371/journal.pone.0015891 (PMC3026797; doi:10.1371/journal.pone.0015891)
Supplement: Table S6 — The clinical and pathological information for the patients of the gastric cancer cohort is reported. For some patients not all information was available (No data). Class: EGC, early gastric cancer; AGC, advanced gastric cancer. (DOC) [file pone.0015891.s007.doc]

**Table S6. Clinical and pathological information of the gastric cancer cohort**

| **Parameter** | **Group** | **GASTRIC COHORT (N=103)** | |
| --- | --- | --- | --- |
|  |  | **N** | **%** |
| **Type** | *Normal* | 23 | 22.3 |
|  | *Metaplasia* | 23 | 22.3 |
|  | *Dysplasia* | 13 | 12.6 |
|  | *Primary tumors* | 31 | 30.1 |
|  | *Metastases* | 13 | 12.6 |
| **Class** | *EGC* | 8 | 25.8 |
|  | *AGC* | 23 | 74.2 |
| **Histotype** | *Intestinal* | 22 | 71. |
|  | *Diffuse* | 9 | 29.0 |
| **Topology** | *Antrum* | 17 | 54.8 |
|  | *Body* | 2 | 6.4 |
|  | *Cardias* | 11 | 35.5 |
|  | *Fundus* | 1 | 3.2 |
| **Grade** | *G1* | 2 | 6.5 |
|  | *G2* | 13 | 41.9 |
|  | *G3* | 16 | 51.6 |
| **pT** | *1* | 8 | 25.8 |
|  | *2* | 12 | 38.7 |
|  | *3* | 10 | 32.3 |
|  | *4* | 1 | 3.2 |
| **pN** | *0* | 13 | 43.3 |
|  | *1* | 5 | 16.7 |
|  | *2* | 8 | 26.7 |
|  | *3* | 4 | 13.3 |
|  | *No data* | 1 |  |
| **Nodal Status** | *Neg* | 13 | 43.3 |
|  | *Pos* | 17 | 56.7 |
|  | *No data* | 1 |  |
